# Radiolabelled ZnO, Iron Oxide-Based, and Gold Nanoparticles for Cancer Therapy: Synthesis, Surface Engineering, and Radiolabelling Strategies

**DOI:** 10.3390/ijms27125299

**Published:** 2026-06-11

**Authors:** Junaid Ali, Albert Comelli, Muhammad Ali, Pierpaolo Alongi, Viviana Benfante

**Affiliations:** 1 Ri.MED Foundation, Via Bandiera 11, 90133 Palermo, Italy; ajunaid@fondazionerimed.com (J.A.); amuhammad@fondazionerimed.com (M.A.); 2 NBFC—National Biodiversity Future Center, 90133 Palermo, Italy; 3 Department of Biomedicine, Neuroscience and Advanced Diagnostics (BiND), University of Palermo, 90127 Palermo, Italy; pierpaolo.alongi@unipa.it; 4 Advanced Diagnostic Imaging-INNOVA Project, Department of Radiological Sciences, A.R.N.A.S. Civico Di Cristina e Benfratelli Hospitals, P.zza N. Leotta 4, 90127 Palermo, Italy

**Keywords:** radiolabelled nanoparticles, zinc oxide nanoparticles, iron oxide-based nanoparticles, gold nanoparticles, chelating agents, radiolabelling, cancer theragnostic, biodistribution, radionuclide therapy

## Abstract

Radiolabelled nanoparticles are increasingly investigated as multifunctional platforms for cancer imaging, biodistribution tracking, dosimetry, and radionuclide-based therapy. This review focuses on three representative inorganic nanoplatforms: zinc oxide (ZnO), iron oxide-based, and gold (Au) nanoparticles. These systems were selected because they combine distinct physicochemical properties with versatile surface engineering and radiolabelling strategies. ZnO nanoparticles offer pH-responsive behaviour and drug-delivery potential; iron oxide-based nanoparticles provide magnetic functionality, Magnetic resonance imaging (MRI) compatibility, and opportunities for magnetic hyperthermia or local nanobrachytherapy; and Au nanoparticles enable stable surface functionalization, radiometal chelation, radiosensitisation, photothermal effects, and alpha or beta-emitter-based local therapy. The review critically discusses synthesis and surface-modification methods, chelator-mediated and chelator-free radiolabelling, coating-assisted and anchoring-mediated strategies, and the influence of these factors on radiochemical stability, biodistribution, tumour uptake, therapeutic response, toxicity, and clearance. A function-based comparison of the reviewed studies highlights that many systems demonstrate efficient radiolabelling and imaging capability, whereas fewer provide direct in vivo therapeutic efficacy, long-term toxicity, or metabolic clearance data. Overall, radiolabelled ZnO, iron oxide-based, and Au nanoparticles show strong potential for cancer theranostics, tumour-to-organ distribution, therapeutic benefit, and safety.

## 1. Introduction

Cancer persists as a major global health burden, representing one of the leading causes of mortality and a formidable challenge in therapeutic intervention. In recent years, the development of targeted therapies has offered new hope by enabling selective treatment of diseased cells while minimizing damage to healthy tissues [1]. Among the emerging platforms under investigation, nanomaterials have attracted significant attention owing to their large surface area, adjustable surface chemistry, and unique physicochemical properties. These attributes render them highly suitable for the delivery of therapeutic radionuclides, offering enhanced control over biodistribution, targeting specificity, and therapeutic efficacy [2]. These materials can integrate their inherent optical, electronic, or magnetic functionalities with the effects of ionizing radiation, enabling the creation of theranostic agents that combine both diagnostic and therapeutic capabilities [3,4]. The selection of appropriate nanomaterial types, radionuclide compound, methods of radionuclide incorporation into inorganic nanosystems, and their efficient delivery to tumor sites are all critical components in advancing cancer treatment strategies [5].

**Figure 1:**
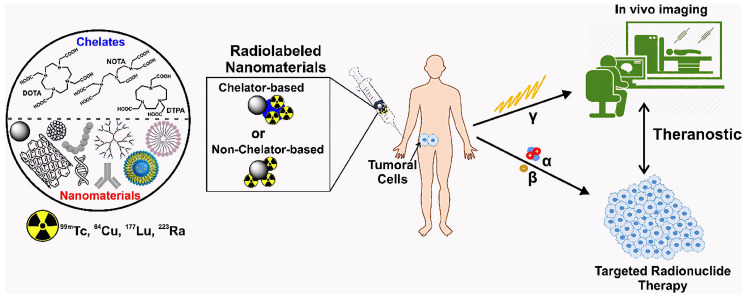
Theranostics process through the utilization of radiolabelled nanomaterials. Reprinted from [37] under CC BY license.

Over the past thirty years, radiopharmaceuticals have established their clinical utility in both cancer diagnosis and treatment (Figure 1). Concurrently, significant progress in nanotechnology has opened up numerous possibilities in the fields of biology and medicine. More recently, the integration of these two areas has led to the development of nanotechnology-enhanced radiopharmaceuticals [6,7]. By leveraging the distinctive structural and functional characteristics of nanoparticles, radiolabeled nanomaterials, often referred to as nano-radiopharmaceuticals, offer promising improvements in diagnostic imaging and therapeutic interventions for various diseases [8]. A broad spectrum of radionuclides is currently synthesized through various production techniques optimized for diagnostic, therapeutic, or theranostic applications, while continuous advancements in delivery systems enhance the precision and therapeutic efficacy of these radiopharmaceutical agents [9,10].

Nuclear medicine has significantly transformed cancer diagnosis and therapy by leveraging radionuclides that emit particulate radiation (such as alpha particles, beta particles, protons, and neutrons) or electromagnetic waves like X-rays and gamma rays [11,12]. When these radionuclides, particularly alpha, beta-minus, or Auger electron emitters are selectively delivered to tumors, they can function as potent therapeutic tools, directly destroying transduced cells through localized radiation [13,14,15,16]. Conversely, radionuclides emitting γ-rays or positrons underpin diagnostic imaging modalities. ^99m^Tc is a notable diagnostic radionuclide, extensively used in SPECT imaging and for labeling nanoparticles in applications such as biodistribution tracking, lymph-node mapping, and the creation of receptor-targeted diagnostic probes. Unless therapeutic goals are clearly measured, ^99m^Tc-labeled iron oxide nanoparticle systems function mainly as diagnostic or tracking tools. To illustrate, ^99m^Tc-labeled PSMA/BN-functionalized iron oxide nanoparticles (IONPs) have been examined for creating SPECT/MRI probes related to prostate cancer and for studying receptor targeting. It is important not to mistake this for therapeutic radionuclide delivery. When captured by tomographic detection systems, these emissions provide accurate spatial mapping of disease sites. Often, isotopic pairs or chemically related radiocompounds are selected such that one isotope supports imaging while its counterpart delivers therapeutic radiation, thereby achieving an intrinsically coordinated theranostic strategy [17,18,19,20]. This dual functionality has fueled rapid innovation in nuclear medicine, driving the continuous design and validation of novel agents. Numerous radionuclide-based compounds for both imaging and treatment have received Food and Drug Administration (FDA) approval and are now routinely used in clinical practice for a wide range of conditions.

Since the early 2000s, nanoscience and nanotechnology have rapidly advanced across scientific and engineering fields, focusing on the development, manipulation, and application of materials at the nanoscale [21,22]. Nanomaterials, engineered at the nanoscale (1–100 nm), exhibit enhanced physical, chemical, and biological properties due to their high surface-area-to-volume ratios, enabling transformative applications across sectors, energy, electronics, and notably in biomedicine [2,23,24,25,26]. The European Technology Platform on Nanomedicine defines nanomedicine as the integration of nanotechnology into medical science [26]. As a result, nanomedicine encompasses the use of nanoparticles (NPs) for various clinical applications like medical imaging, targeted cancer therapy, gene and drug delivery, tissue regeneration, and theranostics (combined diagnostics and therapy). Over time, the field of nanomedicine has advanced significantly, introducing an expanding repertoire of nanomaterials that address previously unresolvable medical challenges. In biological applications, a diverse array of nanoparticles has been explored, such as metallic (e.g., gold, silver), quantum dots, lipids, polymers, dendrimers, and core-shell architectures, allowing precise control over their physicochemical, pharmacokinetic, and biodistribution profiles [27,28]. Advancements in nanomedicine have introduced FDA-approved nanoparticle therapies, which demonstrate superior safety and effectiveness over conventional treatments [29]. These developments address longstanding medical challenges, positioning nanomedicine at the forefront of personalised medicine and next-generation diagnostic and therapeutic strategies [30].

This review specifically focuses on three representative and clinically relevant inorganic nanoparticle platforms: zinc oxide (ZnO), iron oxide (Fe_2_O_3_/SPIONs), and gold (Au) nanoparticles. These systems were selected based on their well-established physicochemical properties, versatility in surface functionalisation, and extensive investigation in radiolabelling and cancer theranostic applications. ZnO nanoparticles offer pH-responsive and drug-delivery potential [31,32]; iron oxide-based systems provide magnetic functionality, MRI compatibility, and opportunities for magnetic hyperthermia or local nanobrachytherapy [33,34]; and Au nanoparticles enable versatile surface chemistry, radiometal chelation, radiosensitization, photothermal effects, and alpha- or beta-emitter-based local therapy [35,36]. By narrowing the scope to these materials, this review aims to provide a more in-depth and comparative analysis of their synthesis strategies, radiolabelling approaches, and therapeutic performance.

Radioactivity is the spontaneous transformation of unstable atomic nuclei into more stable forms, accompanied by the emission of ionising radiation (α, β, or γ). Radiopharmaceuticals harness radionuclides with physical properties (half-life, emission type, and energy) and biochemical properties (targeting and metabolism) suitable for the intended diagnostic or therapeutic use [38,39]. Clinical utility requires matching the radionuclide’s half-life to the carrier’s pharmacokinetics, selecting a suitable emission type (e.g., γ or positron for imaging vs. β or α for therapy), and minimising radiation to non-target tissues [40]. Key quality parameters include radiochemical yield (fraction of initial radioactivity incorporated into the final product), radiochemical purity (percentage of total radioactivity present in the desired chemical form), and radiochemical stability (the radiotracer’s ability to remain intact without decomposition or radiolabel loss over time).

Cancer treatment typically involves a combination of surgery, chemotherapy, and radiotherapy. Radiotherapy utilizes ionizing radiation to induce DNA damage in cancer cells, ultimately leading to cell cycle arrest and programmed cell death [41,42]. It can be delivered externally via X-rays or γ-rays in external beam radiotherapy (EBRT), internally via implanted radioactive seeds (brachytherapy), or systemically via injected radionuclides. Radiotherapy can be used alone or combined with other therapies; however, EBRT lacks tumour selectivity, whereas brachytherapy requires precise tumour localisation. Ionizing radiation causes DNA damage by directly breaking DNA strands or indirectly by generating reactive oxygen species (ROS), resulting in single-strand breaks (SSB) and more lethal double-strand breaks (DSBs) [9,43].

**Figure 2:**
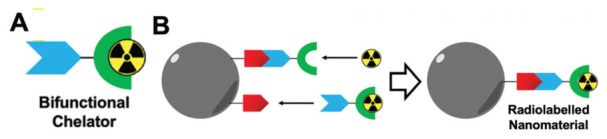
(**A**) Schematic representation of a bifunctional chelator. (**B**) Schematic representation of the radiolabelling of nanoparticles using bifunctional chelators. Reprinted from [36] under CC BY license.

Chelator-based radiolabelling uses chelators covalently attached to nanomaterials to form stable complexes with radiometals like ^64^Cu, ^177^Lu, ^99m^Tc, or ^68^Ga, ensuring in vivo stability. Radiolabeling strategies generally involve two primary approaches: direct chelation, in which the chelating agent is an integral component of the nanomaterial itself, and the bifunctional chelator (BFC) method, wherein the chelator serves as a molecular bridge linking both the nanomaterial and the radionuclide (Figure 2). Non-chelator methods exploit the nanomaterial’s physicochemical properties and include entrapment, direct activation, ion-exchange, and surface adsorption. Entrapment incorporates radionuclides during synthesis, direct activation induces radioactivity by bombardment, ion-exchange swaps lattice ions, and surface adsorption relies on weak interactions. Although non-chelator methods simplify synthesis, they often have lower radiochemical stability than chelator-based approaches [44]. The frequent use of chelating agents in ZnO, iron oxide-based, and Au nanoparticle systems does not negate the potential benefits of chelator-free approaches. Chelator-free or direct incorporation strategies can reduce structural complexity, but they are often material and radionuclide-specific, may require harsh synthesis conditions, and can lead to less predictable radionuclide retention in vivo. In contrast, chelators such as DOTA, NOTA, DTPA, DFO, and TADOTAGA provide modular and relatively mild radiolabelling conditions, high radiochemical purity, improved control over radiometal coordination, and compatibility with targeting ligands, polymers, or drug-loaded nanoplatforms. For these reasons, many reported systems still rely on chelator-mediated or coating-assisted radiolabelling, particularly when preserving nanoparticle size, surface functionality, antibody/peptide activity, and reproducibility is essential.

In this review, “radiolabelling strategy” is used broadly to include both classical chelator-mediated labelling and non-classical radionuclide attachment routes. The reviewed systems are therefore grouped into four main categories: (i) chelator-mediated labelling, (ii) coating-assisted or direct labelling through surface functional groups, (iii) chelator-free core or surface incorporation, and (iv) anchoring-mediated strategies such as Au-S, multithiol, or bisphosphonate-iron oxide interactions. When a cited study does not explicitly use a classical chelating agent, the relevant radionuclide attachment mechanism is described according to the available evidence rather than being generically classified as chelator-mediated labelling.

In vivo stability is not controlled by a single component but by the combined behaviour of the nanoparticle core, surface coating, chelator or anchor, and biological environment. The core affects dissolution and biodegradation; the surface controls colloidal stability, protein-corona formation, circulation time, and RES uptake; and the chelator or anchoring chemistry determines whether the radionuclide remains attached after injection. If radiolabel detachment occurs, radioactivity-based biodistribution may no longer represent the nanoparticle itself. Therefore, true in vivo stability requires integrated evaluation of radiochemical stability, serum challenge, biodistribution, clearance, toxicity, and, where possible, separate tracking of the radionuclide and nanoparticle core. Bifunctional chelators (BFCs) play a critical role in the radiolabeling of nanomaterials by securely coordinating radiometals while covalently attaching to nanoparticles through functional groups such as amines, thiols, or isothiocyanates. This dual functionality ensures the in vivo stability of the radionuclide and preserves the structural integrity of the nanomaterial. Commonly used BFCs, including DOTA, NOTA, and DFO, are specifically optimized for particular radiometals, thereby enhancing both radiolabeling efficiency and overall stability.

The studies are discussed under the three main nanoparticle families: ZnO-based, iron oxide-based, and Au-based systems. Within these sections, the individual studies are interpreted according to their primary function, such as targeted imaging, drug-delivery theranostics, local radionuclide nanobrachytherapy, magnetic hyperthermia-assisted therapy, receptor-targeted imaging/dosimetry, radiolabelling/stability evaluation, or biodistribution studies. A function-based summary is provided in Table 1 to help readers compare studies with similar objectives and distinguish diagnostic, therapeutic, and methodological sections.

## 2. Literature Search and Selection Strategy

**Figure 3:**
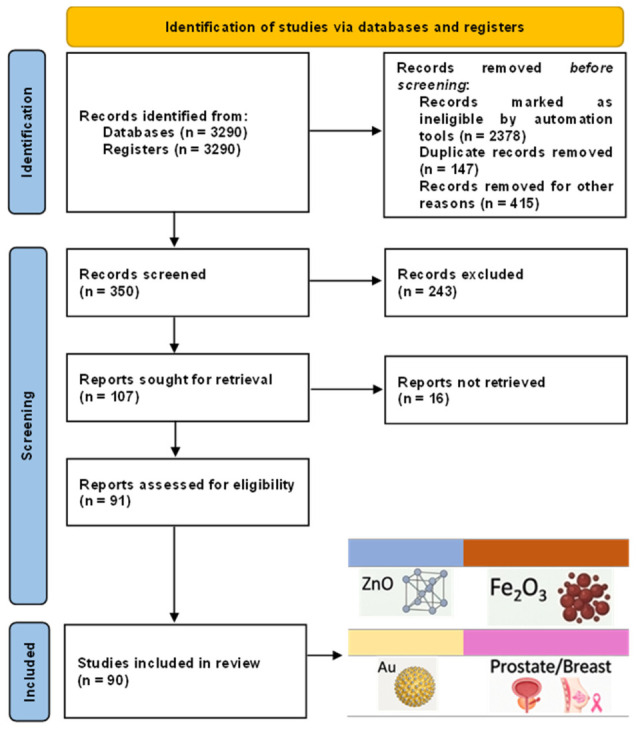
Literature search and selection strategy. Schematic overview of database search, screening, eligibility assessment, and final categorisation of studies included in this review.

A literature search was conducted using Google Scholar, Scopus, PubMed, PubChem, and relevant publisher databases to identify studies on radiolabelled ZnO, iron oxide-based, and Au nanoparticles for cancer imaging, biodistribution, dosimetry, and therapy. The search focused on nanoparticle composition, size, synthesis and surface-functionalisation strategies, radiolabelling approaches, chelating agents or anchoring mechanisms, radiochemical stability, tumour uptake, therapeutic efficacy, toxicity, and clearance. Studies were prioritized when they reported quantitative physicochemical, radiochemical, or biological data relevant to the comparative scope of this review. The analysis considered radionuclides reported in the selected studies ^64^Cu, ^67^Ga, ^68^Ga, ^89^Zr, ^90^Y, ^99m^Tc, ^177^Lu, and ^225^Ac [45,46]. The selected articles were then organized according to nanoparticle composition and primary function, including imaging, biodistribution/dosimetry, radiolabelling/stability, and therapeutic applications (Figure 3).

## 3. Overview of Synthesis Strategies for ZnO, Iron Oxide-Based, and Au Nanoparticles

**Figure 4:**
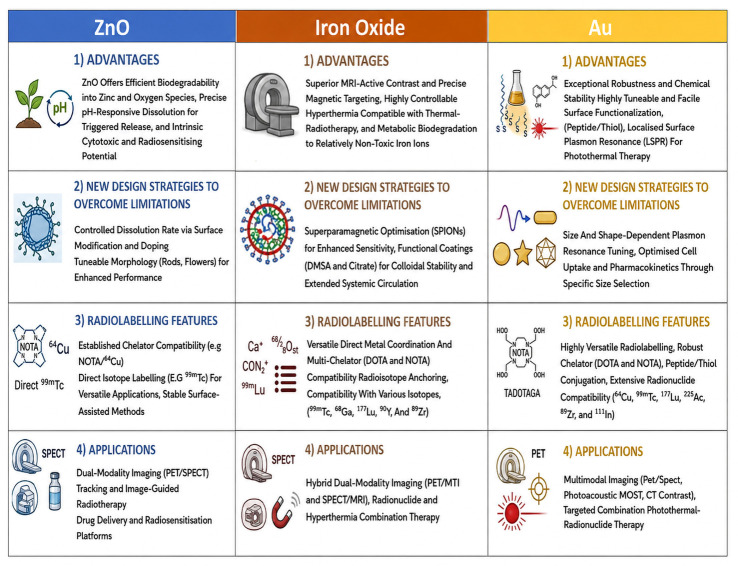
Synthesis of key inorganic nanomaterials for radiothotheranostics.

It is worth emphasising that the synthesis route chosen for inorganic nanoparticles has a direct bearing on their physical and chemical characteristics, including size distribution, morphology, crystallinity, colloidal stability, and surface reactivity, all of which feed into their suitability for radiolabelling and biological use. Hence, synthesis has a central role in determining radiochemical and therapeutic outcomes. ZnO, iron oxide-based, and gold nanoparticles were synthesised via precipitation, sol-gel, biomineralisation, co-precipitation, polyol synthesis, microwave-assisted synthesis, and modified Brust-type reduction. Each method reflects a deliberate choice shaped by the target composition and intended theranostic application (Figure 4).

Several synthesis routes have been explored for ZnO nanoparticles, with precipitation, sol-gel, and vapour deposition being among the most widely applied [47,48]. Precipitation is the most practically accessible of these scalable and relatively easy to carry out, and it allows for further surface modification with polymers like dextran (DEX) or chitosan [49]. Its limitation lies in its sensitivity to reaction conditions: small changes in precursor concentration, pH, temperature, or reaction time can meaningfully affect particle size, aggregation, and surface hydroxylation. Sol-gel auto-combustion can yield more complex, anisotropic morphologies such as nanoflakes, but requires thermal post-treatment and precise control over calcination [47]. Vapour deposition affords the greatest structural control, particularly for nanowire geometries, though it is considerably less amenable to scale-up in a biomedical context [50]. Overall, ZnO synthesis optimised not only for particle formation, but also for surface stability and controlled dissolution, since ZnO degradation under biological or acidic conditions can influence both therapeutic behaviour and radionuclide retention.

Iron oxide-based nanoparticles show the broadest diversity of synthesis routes in the reviewed studies [51,52]. Co-precipitation is one of the most accessible methods for producing Fe_3_O_4_/SPION cores and can be performed under relatively mild aqueous conditions [53], but it often requires careful control of oxidation state, pH, inert atmosphere, and post-synthetic coating to avoid aggregation. Biomineralisation approaches using alginate or related polymers allow simultaneous particle formation and surface stabilisation, generating carboxylate-rich coatings that can support radiometal binding [54,55]. Polyol and solvothermal methods provide better control over crystallinity, magnetic properties, and particle morphology, including iron oxide nanoflowers, but they usually involve higher temperatures and longer reaction times [56]. Microwave-assisted synthesis offers rapid heating and can promote more homogeneous nucleation, but reproducibility depends strongly on power, temperature ramp, and reaction medium.

Au nanoparticles were mainly prepared through citrate reduction, galvanic replacement, and modified Brust-type reduction. Citrate reduction [57] is simple, aqueous, and widely used for producing spherical Au nanoparticles with tuneable size and a negatively charged surface suitable for peptide or thiol conjugation. Galvanic replacement allows the preparation of hollow or cage-like Au nanostructures, such as Au nanocages, with plasmonic properties useful for imaging or photothermal applications. Modified Brust-type reduction provides small, ligand-stabilised Au nanoparticles with strong colloidal stability and surface functionality, particularly when macrocyclic chelators or thiolated ligands are incorporated during or after synthesis [58]. Compared with ZnO and iron oxide-based systems, Au nanoparticle synthesis benefits from robust Au-S surface chemistry, but the non-biodegradable nature of the Au core requires greater attention to particle size, coating density, and long-term clearance.

Across all three material classes, the synthesis method directly affects the subsequent surface-engineering and radiolabelling strategy. Nanoparticles produced with accessible hydroxyl, carboxylate, amine, thiol, or polymer-coated surfaces are generally more suitable for chelator attachment, antibody or peptide conjugation, drug loading, and direct or chelator-free radionuclide binding. Conversely, poorly controlled synthesis can lead to broad particle-size distributions, aggregation, inconsistent coating density, and variable radiolabelling performance. Therefore, future studies should report synthesis parameters more systematically, including precursor concentration, pH, solvent, reaction temperature, reaction time, purification method, particle size distribution, hydrodynamic diameter, zeta potential, crystallinity, and coating density. Such standardisation would allow more meaningful comparison between nanoparticle platforms and would help establish stronger relationships between synthesis route, radiochemical stability, biodistribution, and therapeutic efficacy.

## 4. Zinc Oxide Nanoparticles

**Figure 5:**
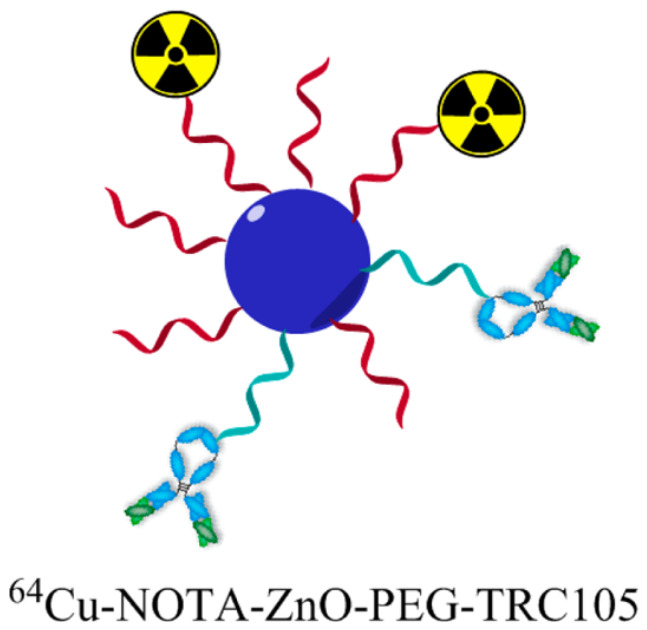
Zinc oxide chelated with NOTA and radiolabelled with ^64^Cu. Reprinted with permission from [59] with slight modification.

H. Hong et al. developed red fluorescent ZnO NPs coupled with TRC105 to target tumours and capture images with PET (Figure 5). Calcination was used to create red luminous ZnO nanoparticles. While stirring, droplets of the zinc acetate dehydrate previously dissolved in dimethylformamide (DMF) were added to the DMF solution that included acetylene dicarboxylic acid and 2-amino terephthalic acid. Zinc 2-amino terephthalate acetylene dicarboxylate coordination polymer produced as yellow precipitates after 10 min of agitation. After filtering and DMF washing, these precipitates were dried for five hours at 60 °C in a vacuum oven. The final product was produced by calcining the dry powders at 550 °C with airflow for 75 min following a heating ramp at two degrees per minute [59].

The zinc oxide surface was functionalised by carboxylic acids to generate bidentate coordination bonds. After adding 3-mercaptopropionic acid, ZnO NPs were suspended in dichloromethane (DCM) and stirred for one hour at room temperature (RT). After DCM evaporated, ethanol was washed from the intermediate product (ZnO-SH) twice. Added to 2 mg of ZnO-SH, maleimide-polyethylene glycol-succinimidyl carboxymethyl ester was incubated at room temperature in phosphate-buffered saline (PBS) for about an hour. Adding tris(2-carboxyethyl)phosphine hydrochloride to the mixture stopped thiol oxidation, therefore maintaining the final pH between 7 and 7.5. The resultant ZnO-PEG-NH_2_ was purified using an Amicon filter; it was further washed using ethanol and water. At pH 8.5, a molar ratio of 1:10 was employed in the two-hour reaction between ZnO-PEG-NH_2_ and S-2-(4-isothiocyanate benzyl)-1,4,7-triazacyclonane-1,4,7-triacetic acid (p-SCN-Bn-NOTA).

**Figure 6:**
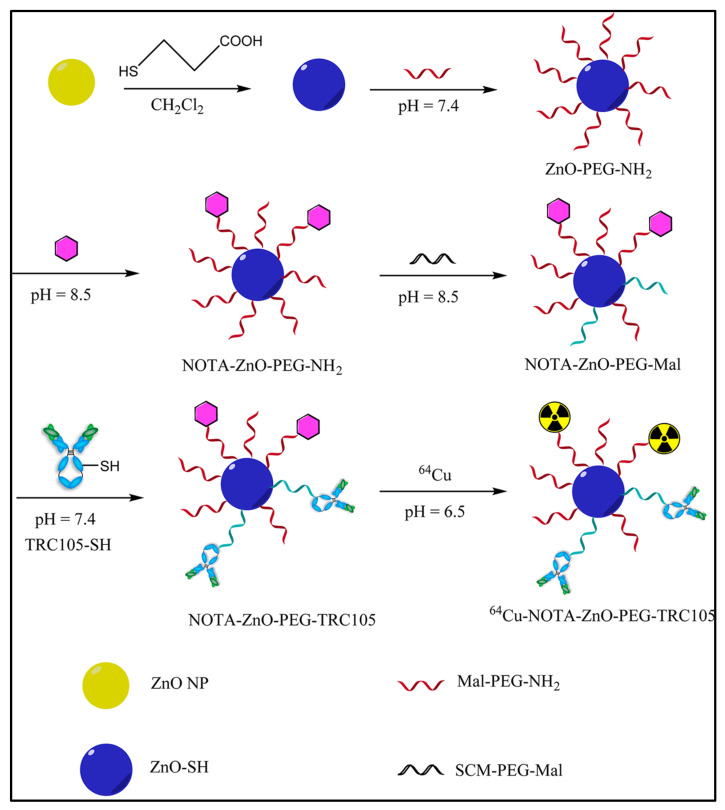
Schematic Synthesis Pathway of NOTA-ZnO-PEG-TRC105 and NOTA-ZnO-PEG-NH_2_. Reprinted with permission from [59].

Once more, the resulting NOTA-ZnO-PEG-NH_2_ was cleaned with an Amicon filter. Another 2-h reaction using NOTA-ZnO-PEG-NH_2_ and succinimidyl carboxymethyl PEG maleimide was conducted at pH 8.5 in a mole ratio of 1:30 using NOTA-ZnO-PEG-Mal. Thiolating TRC105 (TRC105-SH) was accomplished using a previously reported method. After being shielded by TCEP, TRC105-SH then interacted with NOTA-ZnO-PEG-Mal at room temperature (RT) and pH 7.5. NOTA-ZnO-PEG-TRC105, the final product, underwent filtration and centrifugation for purification. Figure 6 provides a demonstration of the entire reaction methods.

Working with sol-gel auto combustion, MH Aboumanei et al. [60] created the chitosan-coated zinc oxide nanoflakes loaded with L-carnitine (CS-LCZnONFS) in order to prevent the chelation among calcium and LC and to achieve therapeutic synergy. Ethylene glycol is added to Zn(NO_3_)_2_·6H_2_O as stated to form ZnO NFS, with a slight modification to the final thermal treatment of the provided ash. The calcined ash at 300 °C is sintered at 400 °C for 3 h using a heating rate of 10 °C/min. LC was dissolved in distilled water at different concentrations to load LC into ZnO NFS. Soon after ZnO NFS addition, the mixture was agitated at 600 rpm for different durations at room temperature. After five minutes of centrifuging the suspension at 5000 RPM, precipitates and supernatant were recovered [60].

The direct labelling method performed radiolabelling of CS-LC-ZnO NFS and LC solution. The correct concentration of the mixture was placed in a penicillin vial, followed by the addition of the necessary concentration of stannous chloride (SnCl_2_·2H_2_O) solution. An amplified mixture was prepared using one milliliter of freshly eluted ^99m^TcO_4_^−^. The study investigated the factors of pH, temperature, reaction duration, substrate level, and SnCl_2_·2H_2_O content that affect the percentage of radiochemical yield. Filtration of the formulations with a 0.2 μm pore sterile filter sterilised them. Ascending paper chromatographic assessment was implemented to assess the radiolabelling efficacy and quality control investigations of ^99m^Tc-CS-LC-ZnONFS and ^99m^Tc-LC solution preparations.

**Figure 7:**
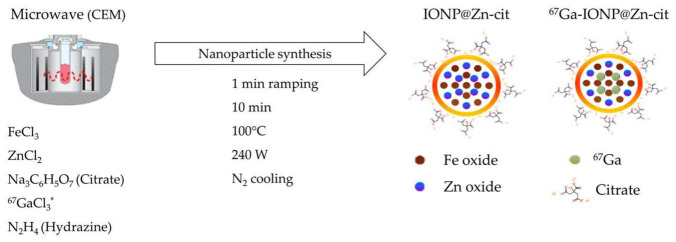
Synthetic scheme of IONP@Zn and their radiolabelling with ^67^Ga. Reprinted from [61] under CC BY license. * In the preparation of ^67^Ga-IONP@Zn-cit, ^67^GaCl_3_ was incorporated into the starting mixture.

In another study, nanoparticle Zn doped iron oxide (IONP@Zinc) developed by M Ibáñez-Moragues et al. [61], stable in serum had with hydrodynamic size of 10 nm (Figure 7). FeCl_3_, ZnCl_2_, trisodium citrate, and 0.5 mL of hydrazine hydrate in H_2_O were amalgamated to synthesise IONP@Zn-cit. After a fast increase to 100 °C over one minute, accompanied by stirring and microwave irradiation at 240 W for ten minutes, the mixture was subsequently cooled with nitrogen gas until it attained 55 °C. These dopped nanoparticles were subsequently refined by gel filtration in water, utilising desalting columns PD-10. ICP-MS was employed to quantify the concentrations of incorporated Fe and Zn [61].

The study employed a Gallium-67 radiolabeled probe, which involved converting ^67^Ga-citrate to ^67^GaCl_3_ via an ion exchange process, thereby enabling the effective incorporation of ^67^Ga into the IONP@Zn-cit structure [62]. Silica Gel MiniSpe-ed™ Cartridges and hydrochloric acid were employed as the eluent. MilliQ water and hydrochloric acid were added to modify the molarity, yielding 1 mL of ^67^GaCl_3_ in 1 M hydrochloric acid. The FeCl_3_, ZnCl_2_, citrate trisodium, and H_2_O milliQ mixture was supplemented with this addition. Finally, under the preceding conditions, hydrazine hydrate was inserted before the entire mixture was exposed to MW irradiation. Purification was done by PD-10 desalting columns and size exclusion chromatography.

Hong et al. [63] demonstrated the application of intrinsically fluorescent zinc oxide (ZnO) nanowires (NWs) for imaging cancer cells Chemical functionalization of the nanowires improved their water solubility, biocompatibility, and decreased their cytotoxicity. A single-zone tube furnace with an alumina tube was used to create ZnO NWs using vapor deposition. The precursor material, ZnO powder, was positioned in the tube’s center. Cooling collars were installed at each end of the tube to create a controlled temperature gradient. A steady argon gas flow of 50 cm^3^/min was maintained, keeping the internal pressure at 300 mTorr. Deposition occurred on a polycrystalline alumina substrate located 20 cm from the precursor, where temperatures reached around 400 °C. The furnace was initially heated to 1000 °C at 40 °C/min over 26 min, then further increased to 1400 °C at 10 °C/min. After maintaining this temperature for 2 h, the system was cooled naturally to room temperature (RT) in an argon atmosphere [63].

A pH-sensitive theranostic platform developed by MM Swidan et al. [64] was composed of dextran immobilised zinc oxide nanoparticles, loaded with doxorubicin (DOX) and radiolabeled with the ^99m^Tc radionuclide (^99m^Tc-labelled DOX-loaded ZnO@dextran). The zinc salt was dissolved in a solvent (ethanol) and subjected to a chemical reaction involving a base sodium hydroxide to precipitate zinc oxide. The reaction conditions, such as temperature and pH, were carefully controlled to ensure the formation of nanoparticles. Upon precipitation, the zinc oxide forms into nanoparticles. Size and morphology are influenced by factors such as the concentration of the precursor, the reaction time, and temperature. Unreacted components and byproducts were eliminated by repeatedly washing the resultant ZnO nanoparticles with pure water. Following washing, the nanoparticles are dried in an oven to produce ZnO powder. Afterwards, a homogeneous solution of dextran(DEX) is formed by dissolving it in water. The dried ZnO nanoparticles were then mixed with the dextran (DEX) solution, allowing the dextran (DEX) to coat the surface of the ZnO nanoparticles, enhancing their stability and biocompatibility. To make sure that the dextran (DEX) has bonded to the ZnO surface, the mixture was agitated for a certain duration of time. This process results in the formation of DOX-loaded ZnO@dextran nanoparticles [64]. Lastly, a radiopharmaceutical kit containing the reagents required to produce ^99m^Tc in a form appropriate for radiolabelling was used to create a ^99m^Tc solution. The ^99m^Tc solution was then added to the DOX-loaded ZnO@dextran nanoparticles. This step ensures that the radionuclide binds effectively to the nanoparticles. The mixture was incubated under specific conditions (temperature and time) to facilitate the binding of ^99m^Tc to the nanoparticles. This process is optimized to achieve high radiolabelling efficiency. After incubation, the final product, ^99m^Tc-labelled DOX-loaded ZnO@dextran, was characterized to confirm successful radiolabelling. This includes assessing radiolabelling efficiency, which was reported to be 98.5%.

High-efficiency magnetic nanoparticles based on zinc- and manganese-doped iron oxide (Zn_0_._4_Mn_0_._6_Fe_2_O_4_) were used by Ni et al. [65] to create magnetically guided nanostructures. These nanoparticles were modified with meso-tetrakis(4-carboxyphenyl)porphyrin (TCPP) and directly labeled with zirconium-89 (^89^Zr), eliminating the need for a chelator. The resulting ^89^Zr-MNP/TCPP complexes enhance tumor-targeted delivery through magnetic navigation, enabling Cerenkov radiation-induced photodynamic therapy (PDT). For nanoparticle synthesis, ZnCl_2_, MnCl_2_, and Fe(acac)_3_ were dissolved in octyl ether within a three-neck round-bottom flask, with oleic acid and oleylamine acting as stabilizing surfactants. The mixture was heated to 300 °C and held for one hour before cooling to room temperature. The precipitated black nanoparticles were then isolated by ethanol addition, centrifugation, and redispersion in chloroform. To enhance hydrophilicity and biocompatibility, the hydrophobic nanoparticles underwent surface modifications with DSPE-PEG5k and DSPE-PEG5k-NH_2_. The MNPs were mixed with these PEGylated lipids in chloroform, briefly stirred, and then subjected to rotary evaporation at 60 °C under vacuum for two hours. Following solvent removal, water was introduced, and the solution was sonicated for 15 min. Excess PEG was removed via centrifugation at 15,000 rpm for 10 min, and the purified PEGylated MNPs were finally suspended in water or PBS [65].

PET imaging was used in a study to investigate the biodistribution and in vivo circulation dynamics of PEGylated magnetic nanoparticles (MNP-PEG). The nanoparticles underwent radiolabeling with zirconium-89 (^89^Zr) utilizing a chelator-free method. For the labeling procedure, MNP-PEG suspended in HEPES buffer was combined with about 1 mCi (37 MBq) of ^89^Zr-oxalate. The solution pH was maintained between 7 and 8 by adding 1 M sodium carbonate (Na_2_CO_3_). This reaction mixture was kept at 75 °C for 2 h with constant agitation. Following the incubation period, the radiolabeled nanoparticles (^89^Zr-MNP-PEG) were separated by centrifugation and subsequently suspended in phosphate-buffered saline (PBS). Autoradiographic imaging was used to ascertain the labeling yield, and thin-layer chromatography (TLC) was used to evaluate radiolabeling effectiveness.

Overall, the ZnO-based systems provide different levels of evidence for cancer imaging and therapy. The ^64^Cu-NOTA-ZnO-PEG-TRC105 platform mainly supports targeted tumour imaging, with CD105-mediated tumour uptake and clear reduction after blocking, but it does not provide therapeutic efficacy data. In contrast, ^99m^Tc-labelled DOX-loaded ZnO@dextran offers the strongest ZnO-based theranostic evidence because it combines high radiolabelling efficiency, pH-responsive DOX release, tumour uptake, and in vivo tumour suppression [64]. The ^99m^Tc-CS-LC-ZnO nanoflake system demonstrates high-yield direct radiolabelling and useful biodistribution tracking; therefore, it should be interpreted as methodological evidence rather than direct cancer-theranostic evidence [60]. Taken together, the ZnO literature remains promising but comparatively limited, and stronger conclusions will require more tumour-bearing efficacy, toxicity, and clearance studies.

## 5. Iron Oxide Nanoparticles

**Figure 8:**
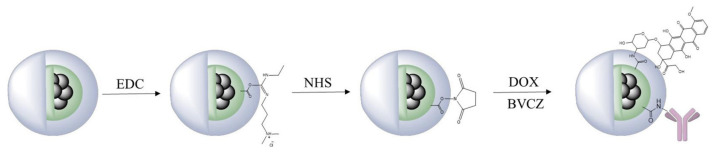
Reaction scheme of the functionalization of magnetic iron oxide nanoparticles (MIONPs) coated with alginic acid and stabilized by polyethylene glycol (MAPEG) with doxorubicin (DOX) and bevacizumab (BVCZ). Reprinted from [66] under CC BY license.

To mitigate breast cancer, EA Salvanou et al. [66] synthesised ^177^Lu-labeled iron oxide nanoparticles (IONPs) functionalised with bevacizumab and doxorubicin (DOX) as nanobrachytherapy agents. The magnetic iron oxide nanoparticles (MIONPs) coated with alginic acid and stabilized by polyethylene glycol (MAPEG) complex was created by coating the initial iron oxide nanoparticles (IONPs) with polyethylene glycol (PEG) and alginic acid. To conjugate doxorubicin (DOX) and bevacizumab (BVCZ) to MAPEG, researchers first chemically activated the carboxyl (-COOH) groups of alginic acid under physiological pH conditions. This activation step utilized N-ethyl-N′-(3-(dimethylamino)propyl)carbodiimide (EDC) and N-hydroxysuccinimide (NHS) as crosslinking agents. For conjugation, bevacizumab and doxorubicin were added, forming covalent connections. Figure 8 illustrates the crosslinking reaction scheme [66].

**Figure 9:**
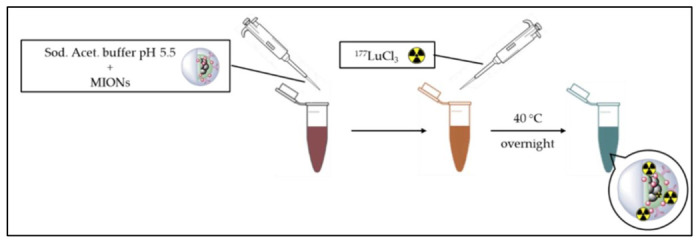
Reaction strategy for the radiolabeling of MIONs with ^177^Lu. Reprinted from [66] under CC BY license.

The nanoparticles were subsequently treated with ^177^Lu in a sodium acetate buffer at pH 5.5. After 30 min of radiolabelling at 75 °C, MAPEG nanoparticles demonstrated 93.65% radiochemical incorporation. For MAPAD (the antibody-functionalized nanoparticles), a lower temperature (40 °C) was maintained to preserve BVCZ immunoreactivity, and radiolabelling was completed overnight, yielding 93.41%. The stability of the radiolabelled nanoparticles was confirmed up to 14 days post-labelling ^177^Lu-labelled nanosystem, as shown in Figure 9.

EA Salvanou et al. [67] performed a preliminary evaluation of iron oxide nanoparticles radiolabelled with ^68^Ga and ^177^Lu, which were evaluated as potential theranostic agents. The synthesis of MA and MAPEG was performed through a biomineralization approach [67]. Magnetic iron oxide nanoparticles (MIONPs) were synthesized using an alkaline precipitation of ferrous sulfate (FeSO_4_·7H_2_O) in the presence of sodium alginate, which provides a polymeric coating. The reaction was conducted at 50 °C for 80 min. Multiple centrifugation steps were carried out to purify MA nanoparticles. The supernatant containing the well-dispersed MA nanoparticles was collected and further functionalized with methoxy-polyethylene glycol-amine (MeO-PEG-NH_2_) to improve their stability and biocompatibility. PEGylation was facilitated by the activation of alginate’s carboxyl groups using coupling reagents such as N, N′-diisopropyl carbodiimide (DIC), hydroxy benzotriazole (HOBt), and N, N-diisopropylethylamine (DIPEA). The reaction was carried out in dimethylformamide (DMF) medium, and excess reactants were removed through repeated centrifugation and washing steps with DMF and ultra-pure water, yielding the final MAPEG nanoparticles. Finally, for radiolabelling, both the MA and MAPEG nanoparticles were incubated with [^177^Lu]LuCl_3_ in sodium acetate buffer (pH 5.4) at 75 °C for 30 min. The carboxyl groups of alginates facilitated the direct binding of ^177^Lu to the nanoparticles, which act as chelation sites for the radioisotope. Instant thin-layer chromatography (ITLC-SG) was used to confirm radiochemical purity, showing high labelling yields (>93%).

A research team led by M. Ognjanovic [68] engineered radiolabeled iron oxide nanoparticles (IONPs) designed for diagnostic use and potential application in combined cancer therapies involving radionuclides and hyperthermia. These nanoparticles were fabricated using a polyol-based approach. In this method, iron salts were precipitated with sodium hydroxide (NaOH) in a mixed solvent system containing diethylene glycol (DEG) and N-methyl diethanolamine (NMDEA). The synthesis begins by dissolving iron (III) chloride hexahydrate (FeCl_3_·6H_2_O) and iron (II) chloride tetrahydrate (FeCl_2_·4H_2_O) in the DEG/NMDEA solvent mixture under continuous stirring for one hour. Next, NaOH was introduced to trigger nanoparticle precipitation, with the reaction proceeding for three additional hours. The mixture then underwent solvothermal processing in an autoclave at 220 °C for 12 h, followed by gradual cooling to ambient temperature [68]. Following precipitation, the iron oxide nanoparticles (IONPs) were magnetically separated and extensively washed with a 1:1 (*v*/*v*) ethanol-ethyl acetate mixture. To eliminate surface defects, the nanoparticles were then subjected to nitric acid (HNO_3_) treatment. This acid etching was followed by the addition of an iron (III) nitrate [Fe(NO_3_)_3_] solution and heating at 80 °C for 45 min to further purify the particles. The resulting nanoparticles were subsequently dispersed in deionized water, filtered, and dried at 60 °C for 24 h. For improved stability and to facilitate radiolabelling, the dried IONPs were surface coated with citric acid (CA), poly(acrylic acid) (PAA), and polyethylene glycol (PEG). Radiolabelling with ^177^Lu was achieved by incubating these functionalized nanoparticles with ^177^LuCl_3_ (0.04 M HCl, pH 5–6) at ambient temperature for 30- or 60-min. Instant thin-layer chromatography (ITLC-SG) analysis indicated a radiolabelling efficiency exceeding 98%. This high yield is attributed to the strong affinity of ^177^Lu^3+^ ions for the carboxylate (–COO^−^) and hydroxyl (–OH) groups on the nanoparticle surface.

Gupta et al. [69] synthesized ^177^Lu-labeled folate radiopharmaceuticals conjugated with iron oxide nanoparticles (IONPs) (^177^Lu-IONP-Folate) to improve overall tissue distribution and reduce the renal absorbed dose. The preparation of ^77^Lu-IONP-Folate was carried out through a series of conjugation and radiolabelling steps. IONP-DBCOs were synthesized by incorporating DSPE-PEG (2000)-DBCO into a 10% (*v*/*v*) polysorbate solution in distilled water, followed by sonication at 30 °C for 30 min. To this micelle mixture, IONPs were added, and the solvent was evaporated using a rotary evaporator. After sonication for 1 h, the reaction mixture was centrifuged and purified at 4000 rpm and 4 °C for 2 h using OptiPrepTM gradients. After purification, the gradient was removed via an Amicon filter. For ^177^Lu-NOTA-DBCO, ^177^LuCl_3_ was added to 1 M sodium acetate buffer (pH 5.6), followed by the addition of NOTA-DBCO. The reaction mixture was incubated at 70 °C for 10 min to generate ^177^Lu-NOTA-DBCO for labelling without purification. Folate-N3 in sodium ascorbate solution was blended into the IONP-DBCO mixture in distilled water at 38 °C for 15 min to yield ^177^Lu-IONP-Folate. A desalting column was used to purify the reaction mixture, and an Amicon filter was used to concentrate it to 100 μL. Lastly, ^177^Lu-NOTA-N_3_ was mixed with IONP-Folate-DBCO and vortexed for one minute at 38 °C [69].

In order to fabricate ^177^Lu-DMSA@SPIONs, D. Stanković et al. [70] coated nanoparticles of superparamagnetic iron oxide (SPIONs) with DMSA and radiolabeled their surface with ^177^Lu. These SPIONs were then assessed in relation to breast 4T1 subcutaneous tumors. The process of co-precipitation was used to create the bare SPION core. Iron (II) sulfate heptahydrate (FeSO_4_·7H_2_O) and iron (III) chloride hexahydrate (FeCl_3_·6H_2_O) were dissolved in 30 mL of deionized water while constantly stirring to form an ion solution. Ammonium hydroxide (NH_4_OH) was added steadily after the solution was heated to 50 °C in order to get the pH down to between 10 and 11. For an additional hour, the reaction mixture was continuously stirred while being kept at 80 °C. The resultant Fe_3_O_4_ nanoparticles (SPIONs) were cleaned with distilled water until the pH stabilized to neutral after being allowed to cool to room temperature and separated by magnetic separation. The SPION suspension (pH~6) was mixed with a DMSA/DMSO solution that functionalized the SPIONs with meso-2,3-dimercaptosuccinic acid (DMSA). This mixture was agitated at room temperature (RT) for 24 h, after which it underwent overnight dialysis in deionized water, with three water exchanges to remove unbound reagents, yielding DMSA@SPIONs. The solution pH stabilized between 7 and 8. The process of radiolabeling DMSA@SPIONs with ^177^Lu involved agitating the nanoparticles with a ^177^LuCl_3_ solution at room temperature for an hour, while 0.1 M NaOH was used to correct the pH. Post-reaction, the ^177^Lu-DMSA@SPIONs were magnetically isolated and rinsed multiple times with deionized water to remove any unbound residues [70].

**Figure 10:**
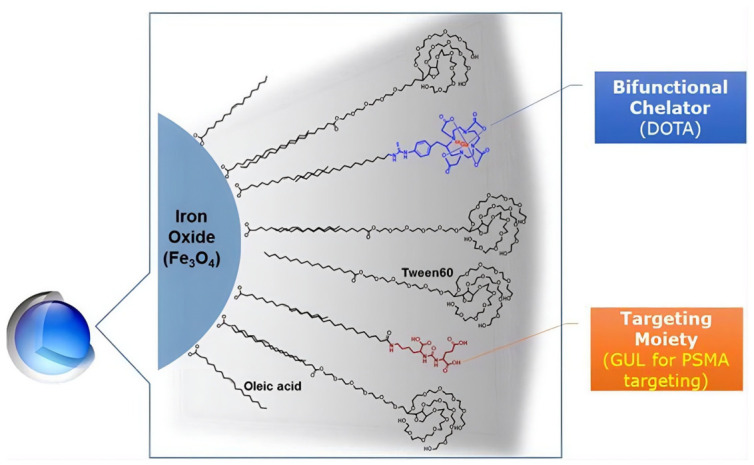
Diagram of encapsulated DOTA-IO-GUL. Reprinted from [71] under CC BY license.

SH Moon et al. [71] developed a new PSMA-targeting IONPs for PET/MR dual-imaging use. The encapsulation method was employed using amphiphiles containing DOTA and GUL, each conjugated with a long alkyl chain and commercially available IONPs (Figure 10). The IO core was used for MR imaging, while the DOTA and GUL moieties were used for labeling with ^68^Ga and targeting PSMA. The DOTA-IO-GUL nanoparticle were prepared through an encapsulation method to functionalize iron oxide (IO) nanoparticles with DOTA and GUL (glutamate-urea-lysine). The synthesis began with the dispersion of DOTA and GUL in a Tween 60 aqueous solution. The solution was sonicated, and iron oxide nanoparticles (IONPs) in chloroform were added gradually. The reaction mixture underwent sonication for 10 min and heating at 80 °C for 10 min, and a cycle was repeated three times to remove chloroform. The solution was subjected to further sonication for 2 h, leading to a final dark brown solution. The product was purified using Sephacryl S-500 HR-packed column chromatography with distilled water as an eluent. The clean brown fractions were collected and concentrated through ultrafiltration. The amalgamation of ^68^GaCl_3_ and 1 M sodium acetate buffer (pH 5.6) was utilised for ^68^Ga radiolabeling. Following the introduction of DOTA-IO-GUL, it was vortexed for one minute. The reaction mixture was incubated at 90 °C for 30 min and thereafter cooled down to room temperature. The labeling efficiency of ^68^Ga-DOTA-IO-GUL was assessed using instant thin-layer chromatography (ITLC). The results demonstrated a radiochemical purity of over 99%, affirming the compound’s stability and appropriateness for PET/MR imaging systems [71].

For dual-modality SPECT/MRI imaging of sentinel lymph nodes (SLNs), R. Madru et al. [72] created biocompatible superparamagnetic iron oxide nanoparticles (SPIONs) radiolabeled with ^99m^Tc. According to TEM, the nanoparticles had a monodisperse Fe_3_O_4_ core that measured 11 nm in diameter and was encased in a functionalized layer of PEG. By reducing clumping and improving biocompatibility, this hydrophilic PEG coating made radiolabeling possible. Using dynamic light scattering (DLS), the iron oxide core and stabilizing PEG shell were both found to have a total hydrodynamic diameter of 18 nm. For radiolabelling with ^99m^Tc, SPIONs were suspended in sterile water. The labelling was performed using ^99m^Tc-pertechnetate ^99m^TcO_4_^−^ at pH 3.4, with stannous chloride (SnCl_2_·2H_2_O) as the reducing agent. First, ^99m^TcO_4_^−^ eluted from a ^99m^Tc generator was injected into a sterile vacuum collection vial. Then, SnCl_2_·2H_2_O was dissolved in distilled water, stirred for a few seconds, and passed through a 0.22-μm Millipore filter into the vial containing ^99m^TcO_4_^−^. The SPION suspension was then added, and the vial was gently shaken for 30 s, followed by incubation at room temperature (RT) for 60 min. The radiochemical stability of the labeled compound was evaluated using instant thin-layer chromatography (ITLC) with silica gel-coated glass fiber sheets and an 85% methanol mobile phase. Radiolabeling efficiency reached 99% within 6 h and demonstrated remarkable stability, retaining 98% integrity in aqueous environments and 97% in human serum after 24-h incubation. These results confirm the compound’s suitability for extended diagnostic or therapeutic applications [72].

**Figure 11:**
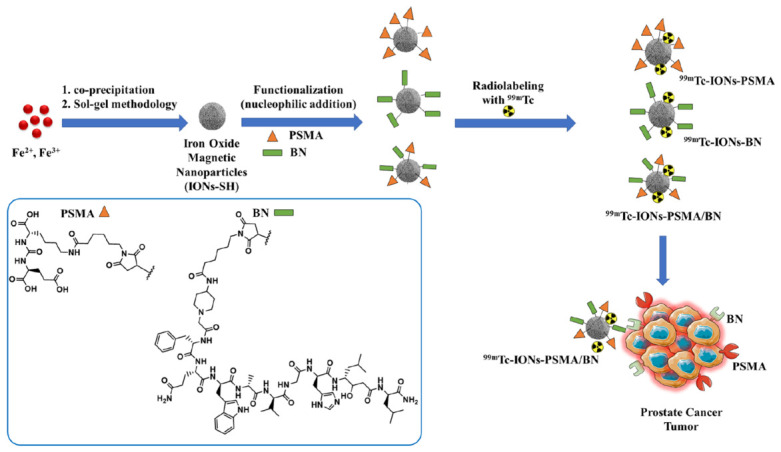
The chemical structures of the IONPs that were radiolabeled with ^99m^Tc and functionalised with the pharmacophores PSMA, BN, or both (PSMA/BN). Reprinted from [73] under CC BY license.

Bajwa et al. [73] developed ^99m^Tc-labelled PSMA/BN-functionalized iron oxide nanoparticles as a prostate cancer-related SPECT/MRI diagnostic platform, rather than as a therapeutic radionuclide system (Figure 11). The iron oxide nanoparticles (IONPs) were functionalized with either PSMA-targeting ligands, bombesin (BN) ligands targeting gastrin-releasing peptide (GRP) receptors, or both ligands to address receptor heterogeneity in prostate cancer. ^99m^Tc was used because of its suitability for SPECT imaging and nanoparticle tracking, while the study mainly evaluated radiolabelling efficiency, serum/challenge stability, biocompatibility, and receptor-associated binding in prostate cancer cell lines. The co-precipitation method was employed to synthesize iron oxide nanoparticles (IONPs) under nitrogen protection. Ammonia (NH_3_) was introduced to a 1:2 molar ratio of Fe^2+^/Fe^3+^ chlorides following the dissolution of ferrous (Fe^2+^) and anhydrous ferric (Fe^3+^) salts under alkaline aqueous conditions at 50 °C. Magnetite (FeO_4_) synthesis involved the dissolution of chloride precursors in deionized water, followed by agitation for 30 min at 70 °C in a nitrogen atmosphere. The reaction mixture was continuously agitated for one hour. After completion, the iron oxide nanoparticles (IONPs) were dispersed in deionized water, purified through three sequential washes of water and ethanol (EtOH), and isolated via magnetic separation. For storage, the final product was refrigerated at 4–6 °C. On the surface of the iron oxide nanoparticles (IONPs), a thin silica layer was created using the sol-gel technique with MPTES. IONs-SH groups were added for additional functionalisation. An ION dispersion was prepared by combining ethanol and water, followed by stirring for 30 min at 80 °C in a nitrogen atmosphere. After adding NH_3_ and MPTES, the reaction mixture was homogenized and left to react for the whole night at 80 °C while stirring continuously. Thiol-functionalized iron oxide nanoparticles (IONPs-SH) were obtained by magnetic separation, refined using ethanol (EtOH) and water in three washing cycles, and then preserved as an aqueous dispersion. Ultimately, the target pharmaceutical molecule was conjugated with IONs-SH [73].

The radiolabelling of IONs with ^99m^Tc was evaluated using two different approaches: one involved direct complexation with the surface of the IONs after Tc (VII) was reduced to Tc (V). The initial method involved direct ^99m^Tc radiolabelling utilising anhydrous SnCl_2_ as the reducing agent. Solution A was created by first dissolving SnCl_2_ in HCl and then adding deionised water. PBS (pH 10) was combined with each nanoparticle variety (IONs-SH, IONs-PSMA, IONs-BN, and IONs-PSMA/BN) separately. After adding Solution A, the pH of this mixture was brought down to 6.5–7.0. Lastly, Na [^99m^Tc]TcO_4_ was added, and it was incubated for an hour at 75 °C. Soon after ^99m^Tc labelling, the IONs were utilized without additional purification, provided their radiochemical concentration (RCC) exceeded 94%.

**Figure 12:**
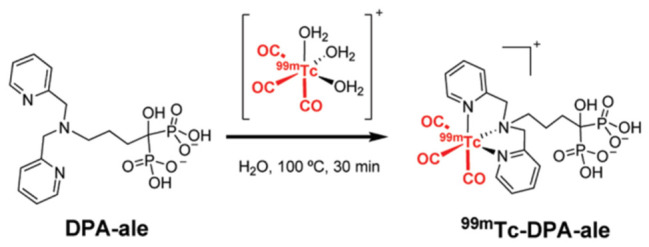
Synthesis of ^99m^Tc-DPA-ale. Reprinted from [74] under CC BY license.

A new class of dual-modality imaging agents based on the conjugation of radiolabelled bisphosphonates (BP) was reported by R Torres Martin de Rosales et al. [74] directly to the surface of superparamagnetic iron oxide (SPIO) nanoparticles. The researchers used ^99m^Tc dipicolylamine (DPA)-alendronate to radiolabel superparamagnetic iron oxide (SPIO) nanoparticles, demonstrating the substantial potential for bisphosphonate (BP)-iron oxide nanoparticle conjugation (Figure 12).

Monodisperse magnetite (Fe_3_O_4_) SPIO nanoparticles lacking any surface coat were synthesized following Stroeve et al. Protocol. To guarantee colloidal stability, uncoated nanoparticles were prepared less than twenty-four hours prior to usage and kept in an aqueous medium that had been nitrogen-purged. A 0.22 μm hydrophilic PTFE membrane was used to filter the nanoparticle dispersion to filter out unwanted aggregation preceding radiolabeling. TEM imaging and optical inspection, however, showed that these uncoated SPIO nanoparticles began to amalgamate in a matter of days, underscoring their short-term stability [74].

**Figure 13:**
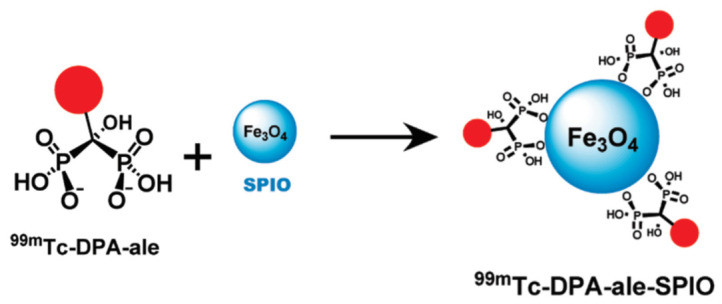
Schematic representation of the synthesis of radiolabeled SPIO nanoparticles ^99m^Tc-DPA-ale-SPIO. Reprinted from [74] under CC BY license.

Standardization techniques were employed in the synthesis and characterization of the ^99m^Tc-DPA-alendronate complex. The procedure involved combining an aqueous [^99m^Tc(CO_3_)(H_2_O)_3_]^+^ solution with DPA-ale dissolved in carbonate buffer (pH 9) in a sealed container. After 30 min of heating at 100 °C, the reaction mixture was quickly cooled in an ice bath. The assessment of radiochemical purity using radio-thin layer chromatography (radio-TLC) and radio-high-performance liquid chromatography (radio-HPLC) yielded a purity of over 99% (Figure 13).

**Figure 14:**
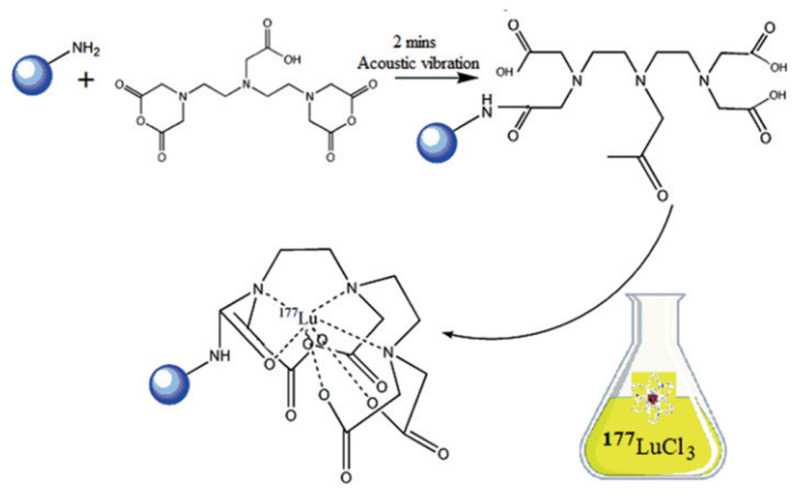
Functionalization and radiolabeling workflow: amine-functionalized dextran-coated iron oxide NPs, conjugation with ccDTPA, ^177^Lu labeling. Reprinted from [76] under CC BY license.

S Shanehsazzadeh et al. [75] reported designing and characterizing a ^177^Lu-DTPA-SPIO as a theranostic radiotracer. The chelator bicyclic anhydride diethylenetriamine penta-acetic acid dianhydride (ccDTPA) was conjugated to SPIO NPs using a minor modification of the well-known cyclic anhydride method. Conjugation was performed at a 1:2 (SPIO:ccDTPA) molar ratio (Figure 14). Briefly, NPs and DTPA anhydride were mixed in phosphate-buffered solution (pH = 9.0) and normal saline and stirred at room temperature (RT) under N_2_ for 1 h. After that, the solution was remixed with an acoustic vibration for 2 min using an ultrasonic processor. Conjugated nanoparticles were purified via high-gradient magnetic separation (MACS) to eliminate unbound fractions [75]. In accordance with the literature, an optimised procedure was used to label the SPIO NPs with ^177^Lu. The protocol began by transferring ^177^Lu-chloride (in HCl) into a conical vial and evaporating the solvent under a nitrogen stream. Phosphate buffer (pH 8) was then introduced to the dried ^177^Lu, followed by the addition of ccDTPA-conjugated nanoparticles (NPs). The mixture was gently agitated for 30 s and incubated at ambient temperature for 45 min. Following incubation, the radiochemical purity of the labeled conjugate was assessed using radio thin-layer chromatography (RTLC) [76].

Vukadinović and collaborators [77] conducted a study evaluating the therapeutic potential of yttrium-90-labeled citric acid-coated superparamagnetic iron oxide nanoparticles (^90^Y-CA/SPIONs) in mouse models of 4T1 breast cancer and CT-26 colon cancer. The SPIONs were fabricated through a polyol-based synthesis approach, where iron salts underwent precipitation with sodium hydroxide in a solvent system containing diethylene glycol (DEG) and N-methyl diethanolamine (NMDEA), following an adapted protocol. Iron salt precipitation was conducted under standard atmospheric conditions in a three-neck round-bottom flask at ambient temperature. The solution was heated incrementally to 220 °C over 3 h, held isothermally for 16 h, and finally allowed to cool within the reaction apparatus [77]. To enhance colloidal stability and incorporate functional groups suitable for radiolabeling, superparamagnetic iron oxide nanoparticles ((SPIONs) underwent surface modification with citric acid (CA), (3-aminopropyl)triethoxysilane (APTES), and dextran (DEX) via standardized protocols. This surface engineering enables the design of targeted and economical radiotherapeutic agents. Subsequent radiolabeling of CA-functionalized SPIONs with ^90^Y adheres to methodologies documented in earlier studies and supplementary resources. The final ^90^Y-CA/SPIONS were then evaluated in vivo therapeutic experiments.

The iron oxide-based systems show the broadest functional diversity among the reviewed nanoparticles, ranging from diagnostic imaging and radiolabelling methodology to local radionuclide therapy and magnetic hyperthermia-assisted treatment. The strongest therapeutic evidence comes from intratumoral nano brachytherapy systems, particularly [^177^Lu]Lu-MAPAD [66], ^177^Lu-DMSA@SPIONs [70], and ^90^Y-CA/SPIONs [77], which showed high local tumour retention and significant tumour-growth suppression. However, these systems performed best after local administration, whereas intravenous delivery generally resulted in liver and spleen sequestration with limited tumour accumulation. Receptor targeted platforms ^68^Ga-DOTA-IO-GUL [71], ^99m^Tc-IONs-PSMA/BN [73], and ^177^Lu-IONP-Folate [69] provide important imaging or dosimetry evidence, but most lack direct therapeutic endpoints. Therefore, iron oxide-based nanoparticles appear most mature for local radionuclide delivery, magnetic-field-assisted strategies, and image-guided applications.

## 6. Gold Nanoparticles (Au NPs)

Y Wang et al. [78] prepared gold nanocages (AuNCs), PEGylated, and radiolabelled with ^64^Cu^2+^ ions. Gold nanocubes (AuNCs) with edge lengths of 55 nm and 30 nm were fabricated by the galvanic replacement method. This approach utilized poly(vinyl pyrrolidone) (PVP)-stabilized silver nanocubes (47 nm and 25 nm edge lengths) as precursors, which were reacted with chloroauric acid to drive nanoparticle formation. The reaction dynamics were analyzed via UV-Vis spectroscopy, where distinct localized surface plasmon resonance (LSPR) peaks confirmed the structural evolution of the nanocubes. For 55 nm AuNCs, the LSPR was adjusted to about 800 nm, while for 30 nm AuNCs, it was around 760 nm. To prepare for ^64^Cu radiolabeling via DOTA-NHS-ester conjugation, gold nanocubes (AuNCs) were first functionalized with NH_22_-PEG-SH. Purified AuNCs were incubated with NH_22_-PEG-SH at 4 °C overnight under continuous agitation. Unbound NH_22_-PEG-SH was eliminated through centrifugation (12,000 rpm, 8 min), and the PEGylated AuNCs were isolated by washing five times with ultrapure water. Trace metal was eliminated by prechelexing the PEGylated AuNCs before they were reconstituted in phosphate buffer (pH 7.4). The solution was mixed with DOTA-NHS-ester (DOTA/AuNC) and reacted at 20 °C for 1 h, followed by thorough centrifugation at 12,000 rpm for 8 min and washing with ultra-purified water to remove the unconjugated DOTA-NHS-ester and to obtain DOTA-PEG-AuNCs [78].

**Figure 15:**
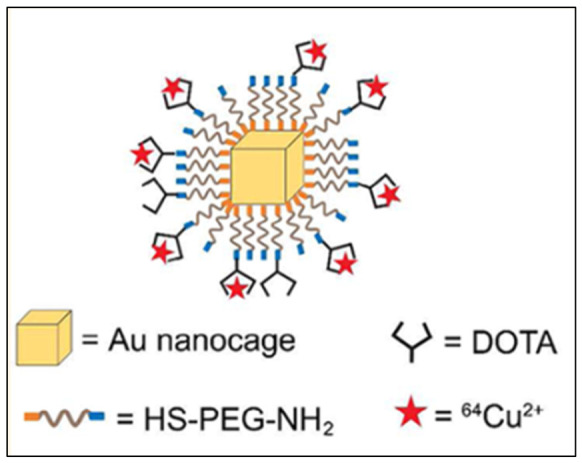
Gold nanocages (AuNCs) functionalized with DOTA-NHS-ester radiolabelled with ^64^Cu. Reprinted with permission from [78].

Optimal radiolabeling efficiency for DOTA-PEG-AuNCs was achieved by reacting the nanoparticles with ^64^Cu in a sodium acetate buffer (pH 7.0) at 55 °C for 1 h (Figure 15). Post-labeling, the ^64^Cu-DOTA-PEG-AuNCs underwent an EDTA stability test in a phosphate buffer and were purified through five ultracentrifugation cycles (12,000 rpm, 8 min per cycle). The radiochemical purity of the final ^64^Cu-DOTA-PEG-AuNCs was validated using radio-thin layer chromatography (radio-TLC).

Yang et al. [79] engineered a uniform hetero-nanostructure by integrating both gold (Au) and iron oxide (Fe_3_O_4_) nanomaterials within a single architecture. This affibody-functionalized trimodal imaging nanoprobe was radiolabeled with copper-64 (^64^Cu), enabling its use in advanced multimodal diagnostic applications. Au-IONPs (gold-iron oxide nanoparticles) synthesis and their surface modification involved a multi-step process. Initially, the Au-IONPs were synthesized following a modified procedure, where iron pentacarbonyl was injected into a reaction mixture under controlled conditions.

The heating rate, set at 8 °C per minute, was crucial in achieving well-defined dumbbell-shaped nanoparticles with a narrow size distribution. The resulting nanoparticles comprised gold (Au) and iron oxide (Fe_3_O_4_) components, which provided optical and magnetic properties, respectively.

To enhance stability and dispersibility in aqueous solutions, the nanoparticles surface was coated with polyethylene glycolate (PEGylated) dopamine linkers, which not only prevented aggregation but also provided amine functional groups for further conjugation. The Au surface was modified with thiolated PEG via Au-S bonds, while the Fe_3_O_4_ component served as an attachment point for biomolecules.

**Figure 16:**
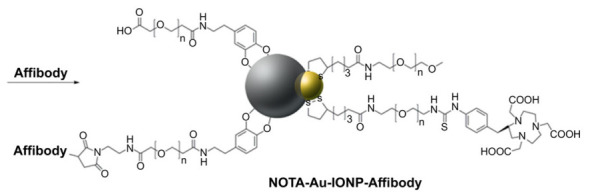
The structure of the affibody attached with NOTA-Au-IONS. Reprinted with permission from [79] with slight modifications.

For the preparation of NOTA-Au-IONP Mal, the Au-IONPs were activated using EDC (1-ethyl-3-(3 dimethylaminopropyl)carbodiimide) and sulfo-NHS (N-hydroxysulfosuccinimide) to introduce maleimide functional groups. After purification through dialysis, these maleimide-activated Au-IONPs were reacted with functional PEG linkers, including LPA-PEG-2000-NOTA and LPA-mPEG-2000, allowing for efficient conjugation of a chelating agent to the nanoparticle surface (Figure 16). This modification facilitates the subsequent radiolabelling process [79].

For radiolabelling with ^64^Cu, the NOTA-functionalised Au-IONPs were mixed with ^64^CuCl_2_ in a sodium acetate buffer (pH 6.0) and incubated at 40 °C for 1 h. The radiolabelled nanoparticles were purified using a PD-10 desalting column, and the radiochemical yield was typically around 60%. The purified ^64^Cu-NOTA-Au-IONP nanoparticles were evaluated for stability by incubating them in mouse serum at 37 °C for up to 24 h, with periodic assessments of retained radioactivity. Successful radiolabelled and stability of ^64^Cu-NOTA-Au-IONPs make them suitable for multimodal imaging applications, including PET, MRI, and optical imaging in tumour-targeting studies.

Sakr and collaborators engineered a novel theranostic nano-radiopharmaceutical by functionalizing gold nanoparticles (AuNPs) with doxorubicin (DOX) and radiolabeling them with ^99m^Tc, designated as ^99m^Tc-Dox-EGCG-AuNPs. The synthesis of Epigallocatechin gallate-coated gold nanoparticles (EGCG-AuNPs) utilises an eco-friendly method, combining EGCG solution with sodium tetrachloroaurate dihydrate (NaAuCl_4_·2H_2_O). Nanoparticle development was confirmed by visible ruby-red shading, signalling the optimal nanosized particle generation. The colloidal suspension was subsequently diluted with a biocompatible physiological solution to enhance stability. For drug loading, DOX was adsorbed onto the EGCG-AuNPs surface via an electrostatic adsorption process, driven by interactions between the positively charged amine groups of DOX and the negatively charged phenolic moieties of EGCG [80]. Additionally, hydrophobic interactions further facilitated DOX attachment to the nanoparticle surface. This stable electrostatic binding ensured that the Dox-EGCG-AuNPs maintained an appropriate nano-size (10–100 nm) for effective tumour penetration via the Enhanced Permeability and Retention (EPR) effect. The optimised formulation involved mixing doxorubicin solution with EGCG-AuNPs, ensuring high drug loading without compromising nanoparticle stability.

For radiolabelling with ^99m^Tc, doxorubicin (DOX) was first labelled using ^99m^Tc-pertechnetate (^99m^TcO_4_^−^) and stannous chloride (SnCl_2_) as the reducing agent. The optimal conditions for radiolabelling were 1 mg of doxorubicin, 100 µg of SnCl_2_, reaction time of 15 min at room temperature, and pH 7, achieving a radiochemical yield of 93.5 ± 2.04%. Following successful labelling, ^99m^Tc-Dox-EGCG-AuNPs were prepared by mixing the radiolabelled ^99m^Tc-doxorubicin (DOX) solution with EGCG-AuNPs under magnetic stirring for 20 min. The final ^99m^Tc-Dox-EGCG-AuNPs were evaluated for stability in saline and rat serum, maintaining radiochemical integrity for up to three days, confirming their suitability for in vivo applications in tumour imaging and therapy.

Jiménez-Mancilla and colleagues investigated the efficacy of a plasmonic photothermal therapy combined with targeted radiotherapy using dual-labeled gold nanoparticles (^99m^Tc/^177^Lu-AuNP-Tat-BN) in PC3 prostate cancer cells. Citrate-stabilized gold nanoparticles (5 nm diameter, AuNP) were commercially sourced and employed without modification. Peptides-Tat-BN, HYNIC-TOC, and DOTA-GGC-were dissolved in injectable-grade water for conjugation. For nanoparticle functionalization, 0.025 mL aliquots of each peptide solution were introduced into 1 mL of AuNP suspension under agitation for 5 min. Thiol-mediated spontaneous binding enabled approximately seven peptide molecules to attach per nanoparticle. The ternary conjugate (DOTA-GGC-AuNP-HYNIC-TOC-Tat-BN) was synthesized by simultaneously incorporating all three peptides. Quantification of peptide loading was validated through UV-Vis spectroscopic titration [81]. To prepare the dual-labeled ^99m^Tc/^177^Lu-AuNP-Tat-BN nanoparticles, 0.02 mL of Tat-BN was initially introduced to 1 mL of AuNP solution. Subsequently, 0.05 mL aliquots of ^177^Lu-DOTA-GGC and ^99m^Tc -HYNIC-TOC were supplemented to the mixture. After continuous agitation for five minutes, radiolabeled nanostructures were formed. Radiochemical purity analysis via size-exclusion chromatography and ultrafiltration demonstrated a labeling efficiency of 96 ± 2% for the final product.

**Figure 17:**
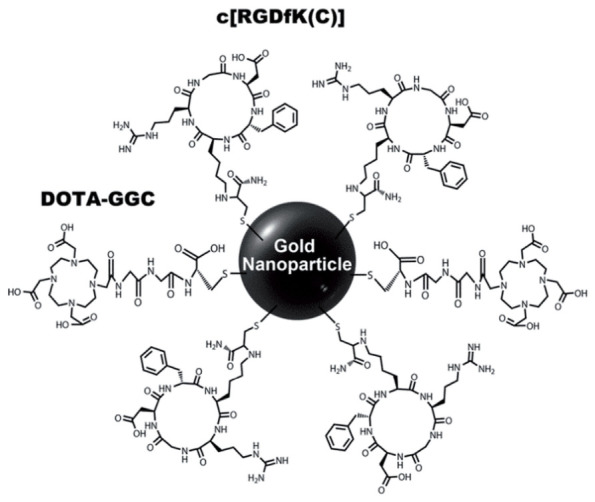
Chemical structure of DOTA-GGC-AuNP-c[RGDfK(C)]. Reprinted from [82] under CC BY license.

Luna-Gutiérrez and colleagues investigated the efficacy of dual-modality cancer therapy using ^177^Lu-labeled gold nanoparticles functionalized with cyclo-[RGDfK(C)] peptides (^177^Lu-AuNP-c[RGDfK(C)]). This system combined plasmonic photothermal and targeted radiotherapy effects on MCF7 breast cancer cells. The gold nanoparticles (AuNPs) were fabricated via a citrate-mediated reduction process. In this synthesis, a boiling trisodium citrate dihydrate solution was prepared under reflux with constant agitation. A 1% tetrachloroauric acid (HAuCl_4_.3H_2_O) solution was subsequently introduced, initiating a sequence of color transitions from pale yellow to black, ultimately stabilising as a deep red, indicative of AuNP synthesis. The colloidal solution was purified by dialysis with injectable-grade water (two water exchanges over a 6-h period), and then vacuum evaporation was used to concentrate it to 20% of its initial volume. Finally, the AuNPs were sterilized via membrane filtration (0.22 µm, Millipore, Darmstadt, Germany). The nanoparticles’ optical properties were analyzed using UV-Vis spectroscopy, confirming the characteristic plasmon resonance band at 522 nm. The peptides DOTA-GGC (1,4,7,10-tetraazacyclododecane-N′,N″,N′′′-tetraacetic-Gly-Gly-Cys) and c[RGDfK(C)] (cyclo[Arg-Gly-Asp-Phe-Lys(Cys)]) were synthesized as reported previously [8,10]. In the c[RGDfK(C)] peptide, the -RGD- sequence functions as the active biological site, while D-Phe (f) and Lys (K) complete the cyclic and pentapeptide structure. The Cys (C) residue serves as the active thiol group, enabling interaction with the gold nanoparticle surface. The Cys residue helps nanoparticles adhere to DOTA, the GG sequence works as a spacer, and DOTA chelates lutetium-177 in DOTA-GGC (Figure 17). Injectable-grade water was used to dissolve c[RGDfK(C)] or DOTA-GGC in order to conjugate peptides to the nanoparticles. One milliliter of a 20 nm AuNP suspension was combined with 0.02 mL of this solution, and the mixture was agitated for 5 min. Approximately 172 peptide molecules are attached to each AuNP under such conditions. Since the maximal binding capacity of 20 nm AuNPs is between 520 and 1701 molecules, no further purification was required. The peptide-nanoparticle ratio, critical for forming DOTA-GGC-AuNP-c[RGDfK(C)], was validated using UV-Vis titration [82].

The synthesis of ^177^Lu-DOTA-GGC involved combining 15 µL of DOTA-GGC with 50 µL of acetate buffer (pH 5) and 7 µL of ^177^LuCl_3_ solution. The mixture was heated at 90 °C for 30 min, after which it was adjusted to a final volume of 1.5 mL using injectable water. Radiochemical purity, confirmed to exceed 98%, was verified via thin-layer chromatography (TLC) and reverse-phase HPLC (RP-HPLC). For the ^177^Lu-AuNP-c[RGDfK(C)] complex, 1 mL of AuNP solution was first functionalized with 0.025 mL of c[RGDfK(C)] solution. To this mixture, 0.025 mL of ^177^Lu-DOTA-GGC was added, followed by 5 min of continuous stirring to ensure nanoparticle-ligand assembly.

**Figure 18:**
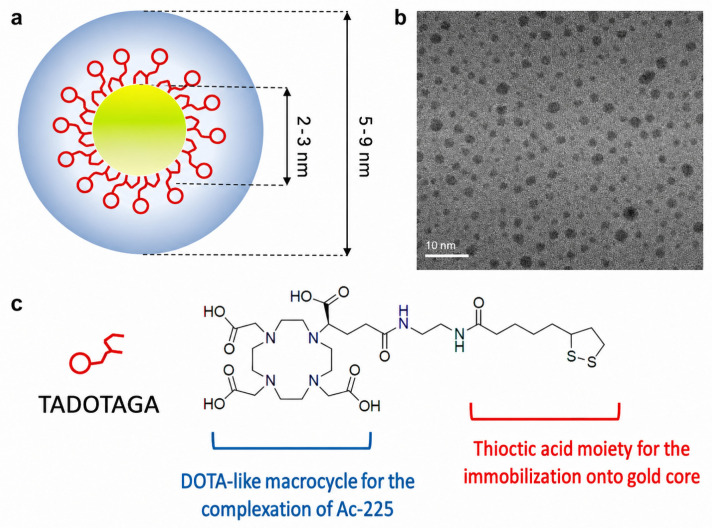
(**a**) Schematic of Au@TADOTAGA structure: Gold core (yellow), TADOTAGA shell (red), and solvation layer (blue). (**b**) TEM image showing Au@TADOTAGA nanoparticle morphology. (**c**) Chemical structure of the TADOTAGA ligand. Reprinted from [83] under CC BY license.

Salvanou and collaborators engineered a novel brachytherapy (BRT) agent by radiolabeling gold nanoparticles with ^225^Ac via a DOTA-based chelator, designed for localized cancer radiation therapy. The macrocycle-coated nanoparticles (Au@TADOTAGA) were synthesized using the modified Brust method, a single-phase reduction synthesis that ensures uniform particle size and enhanced colloidal stability. In the procedure, 50 mg of HAuCl_4_·3H_2_O was first dissolved in 20 mL of methanol before being transferred into a 250 mL round-bottom flask for subsequent reaction steps. In parallel, a solution containing 86 mg of TADOTAGA (Figure 18a) dissolved in 10 mL of water was gradually introduced into the gold salt mixture under continuous agitation. This triggered a chromatic shift from yellow to orange, confirming the formation of gold coordination complexes. A sodium borohydride (NaBH_4_) aqueous solution, prepared by dissolving 48 mg of the reagent in 3 mL of water, was gradually introduced into the reaction mixture under vigorous agitation at room temperature. The reduction reaction was maintained for one hour to ensure complete nanoparticle formation. Post-synthesis, unbound reactants were removed via dialysis using a membrane with a 6–8 kDa molecular weight cut-off (MWCO), yielding purified Au@TADOTAGA nanoparticles (Figure 18b). The obtained nanoparticles were confirmed to have high colloidal stability, with their core sizes ranging between 2–3 nm and the entire nanoparticle complex, including the organic shell, measuring between 5–9 nm (Figure 18c) [83].

The radiolabelling of Au@TADOTAGA with Actinium-225 was performed under optimized conditions to incorporate the radionuclide efficiently. A solution of Au@TADOTAGA nanoparticles (100 µL, containing 5.07 nmol of gold) was prepared in a trace-free sodium acetate buffer (0.2 M, pH 5.6). To this solution, 10–50 kBq of ^225^AcCl_3_ was added, followed by gentle vortexing. The mixture was then incubated at 70 °C for 30 min to facilitate the radiochemical reaction. After cooling to room temperature (RT), the efficiency of ^225^Ac incorporation into the nanoparticles was assessed using instant thin-layer chromatography (ITLC). To enhance the purity of the radiolabelled product, the sample underwent centrifugation at 8110 rcf for 15 min using Amicon Ultra 2 mL centrifugal filters. Post-purification, the radiochemical purity of ^225^Ac-Au@TADOTAGA exceeded 93%, confirming the successful labelling process.

**Figure 19:**
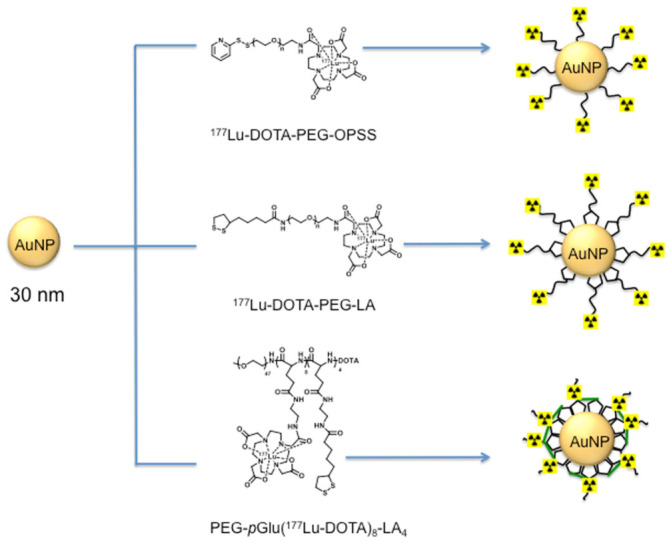
Three functional ligands, ^177^Lu-DOTA-PEG-OPSS, ^177^Lu-DOTA-PEG-LA, and PEG-pGlu(^177^Lu-DOTA)_8_-LA_4_, were reacted with 30 nm gold nanoparticles (AuNPs) under controlled conditions (16 h at 4 °C) to produce the ^177^Lu-MCP-AuNP complex. Reprinted from [84] under CC BY license.

S. Yook et al. [84] studied the stability of AuNP conjugated to MCP via a single thiol [DOTAPEG-ortho-pyridyl disulfide (OPSS)], a dithiol [DOTA-PEG-lipoic acid (LA)] or a multithiol end-group [PEG-pGlu(DOTA)_8_-LA_4_] and determined the elimination and biodistribution of these ^177^Lu-labeled MCP–AuNP in mice (Figure 19). The synthesis of ^177^Lu-DOTA-PEG-OPSS-AuNP, ^177^Lu-DOTA-PEG-LA, and PEG-pGlu(^177^Lu-DOTA)_8_-LA4 followed a common preparation strategy involving the functionalization of gold nanoparticles (AuNPs) with polyethylene glycol (PEG) derivatives, enabling efficient conjugation with DOTA (1,4,7,10-tetraazacyclododecane-1,4,7,10-tetraacetic acid) chelators for radiolabelling with Lutetium-177 (^177^Lu). The related PEG-metal chelating polymers (MCP) were incubated with gold nanoparticles (30 nm in diameter) in low-binding microcentrifuge tubes under carefully monitored conditions. The MCPs DOTA-PEG-OPSS, DOTA-PEG-LA, and PEG-pGlu(DOTA)8-LA4 supplied varying quantities of thiol-based anchoring groups (mono, di, and multi) for conjugation to the AuNP surface. The reaction was carried out at 4 °C for 16 h to allow for efficient surface modification of the AuNPs by the MCPs, forming MCP–AuNP conjugates. The conjugation efficiency was confirmed by UV-Vis spectroscopy and dynamic light scattering (DLS), showing an increase in hydrodynamic diameter after functionalization. The functionalized MCP-AuNP complexes were purified by ultracentrifugation twice at 10,000× *g* for 15 min at 4 °C after conjugation to eradicate any unbound MCPs. For additional radiolabelling, the cleaned nanoparticles were reconstituted in deionized water [84].

The radiolabelling of all three formulations followed a standardized protocol, ensuring high incorporation efficiency of Lutetium-177 (^177^Lu) into the DOTA-functionalised AuNPs. Each MCP-AuNP formulation (DOTA-PEG-OPSS-AuNP, DOTA-PEG-LA-AuNP, or PEG-pGlu(DOTA)_8_-LA4-AuNP) was mixed with 177LuCl_3_ in 0.1 M sodium acetate buffer (pH 6.0). The reaction mixture was incubated at 80 °C for 20 min to facilitate the complexation of ^177^Lu into the DOTA chelating sites. The radiolabelled nanoparticles (^177^Lu-MCP-AuNP) were then purified via ultracentrifugation to remove any unbound 177Lu-MCP. The final radiochemical purity was greater than 99%, as confirmed by thin-layer chromatography (TLC). These optimized ^177^Lu-MCP-AuNP formulations were then characterized for stability, biodistribution, and in vivo elimination, confirming their potential for targeted radionuclide therapy in cancer treatment.

The Au nanoparticle studies differ substantially in translational maturity. Imaging-focused systems like ^64^Cu-DOTA-PEG-Au [78] nanocages and ^64^Cu-NOTA-Au-IONP-Affibody [79] demonstrate how PET or multimodal imaging can guide nanoparticle design, tumour localisation, and receptor-specific targeting, but they do not establish therapeutic benefit. The ^177^Lu-AuNP-c(RGDfK)C system provides stronger dosimetry support by showing improved tumour residence time and absorbed dose compared with monomeric and dimeric RGD analogues, although direct tumour-growth inhibition was not tested [82]. Among the Au systems, [^225^Ac]Ac-Au@TADOTAGA provides the most direct in vivo therapeutic evidence, with tumour-growth delay and histopathology-confirmed necrosis after intratumoral administration [83]. In contrast, ^99m^Tc/^177^Lu-AuNP-Tat-BN [81], ^99m^Tc-Dox-EGCG-AuNPs, and ^177^Lu-MCP-AuNPs [84] are better interpreted as in vitro, preliminary biodistribution, or radiolabelling-stability studies. Overall, Au nanoparticles are highly versatile for radiolabelling, imaging, dosimetry, and local therapy, but systemic therapeutic translation still requires improved radionuclide retention, tumour-selective accumulation, RES avoidance, and long-term safety validation.

To enable a more consistent comparison between the reviewed nanoparticle systems, the available data was reorganised using a unified framework. For each ZnO, iron oxide-based, and Au nanoparticle system, the comparison includes, where reported, the synthesis or surface-engineering strategy, particle size or hydrodynamic diameter, zeta potential, drug or ligand loading/encapsulation efficiency, radiolabeling yield or radiochemical purity, in vitro stability, biodistribution or tumor uptake, therapeutic efficacy, toxicity, and clearance-related observations. Parameters that were not provided in the original studies are indicated as “NR” rather than inferred. This standardised structure allows readers to compare the strengths and limitations of different nanoparticle systems more directly and avoids overinterpreting studies that report only partial physicochemical or biological data.

The reviewed literature shows that efficient labelling and imaging do not automatically imply therapeutic applicability. Many systems remain at the stage of radiochemical feasibility, biodistribution, or tumour visualisation, while fewer provide direct evidence of tumour-growth inhibition, toxicity, organ dosimetry, or metabolic clearance. This gap is important because poor tumour retention, RES accumulation, radiolabel detachment, or delayed clearance can limit clinical translation even when radiolabelling efficiency is high. Among the reviewed systems, stronger therapeutic evidence was observed mainly for locally administered platforms, including ^99m^Tc-labelled DOX-loaded ZnO@dextran, [^177^Lu]Lu-MAPAD, ^177^Lu-DMSA@SPIONs, ^90^Y-CA/SPIONs, and [^225^Ac]Ac-Au@TADOTAGA. Therefore, therapeutic claims should be restricted to systems with direct in vivo efficacy and safety evidence, while imaging or biodistribution only studies should be described as preliminary or diagnostic platforms.

**Figure 20:**
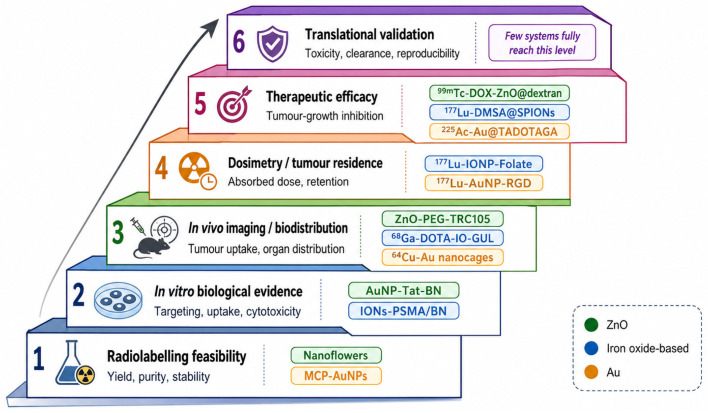
Translational maturity of radiolabeled nanoparticle systems.

The reviewed systems differ substantially in their level of experimental validation. Some studies demonstrate only radiolabelling feasibility, colloidal stability, or in vitro uptake, whereas others provide in vivo imaging, biodistribution, dosimetry, tumour residence, or therapeutic efficacy data. Only a limited number of systems reach the level of in vivo tumour-growth inhibition, and even fewer provide complete toxicity, clearance, reproducibility, and long-term safety evaluation. This evidence highlights the need to distinguish between diagnostic imaging platforms, preliminary therapeutic concepts, dosimetry-supported systems, and truly validated therapeutic nanomedicines Figure 20.

## 7. Conclusions and Summary

A major advancement in direct and indirect radiolabeling approaches for inorganic nanoparticles has been made due to improved radionuclide stability and minimizing radioisotope release into solvents, as discussed in this review. Although chelator-free and direct incorporation methods can simplify nanoparticle architecture, current evidence shows that chelator-mediated and coating-assisted strategies remain dominant because they offer broader radionuclide compatibility, milder labelling conditions, and more predictable radiochemical control. Future development should therefore not treat chelator-free and chelator-based methods as competing approaches but rather select the radiolabelling strategy according to nanoparticle composition, radionuclide chemistry, intended route of administration, and required in vivo stability. At the same time, inorganic materials may be improved by way of intrinsic radiolabeling, method robustness will be enhanced, and the number of chemical reactions will be reduced.

All three types of nanoparticles (ZnO, iron oxide based, and Au NPs) were radiolabeled with high yields via chelators. AuNPs also uniquely support intrinsic radiolabeling (e.g., incorporating ^198^Au into the gold lattice). Using strong chelators matched to the radionuclide (such as DOTA for ^177^Lu or NOTA for ^64^Cu) ensures labeling remains stable. In contrast, inadequate chelation stability or nanoparticle degradation may result in radionuclide dissociation, exemplified by partial ^64^Cu release upon ZnO nanoparticle dissolution in vivo. Inorganic nanocarriers are generally biocompatible with proper surface engineering, but each material has distinct considerations. AuNPs are chemically inert and generally exhibit minimal acute toxicity; however, as gold is non-biodegradable, AuNPs can accumulate and persist in organs such as the liver and spleen. In contrast, superparamagnetic iron oxide nanoparticles (SPIONs) undergo partial biodegradation into iron ions and are typically well tolerated, with several iron oxide formulations approved by the FDA. Nonetheless, excessive superparamagnetic iron oxide nanoparticles doses may induce oxidative stress or inflammatory responses. ZnO NPs dissolve entirely into Zn^2+^ ions, which favors clearance; however, rapid ZnO dissolution can generate reactive oxygen species (ROS) and be cytotoxic at high concentrations. With appropriate coatings and dosing, all three nanoparticle types have shown significant in vivo tolerability.

All three nanoparticle platforms can be directed toward tumor tissues through both passive targeting, mediated by the enhanced permeability and retention (EPR) effect, and active targeting, achieved via ligand-receptor interactions. AuNPs are easily functionalized with tumor-specific ligands (e.g., PSMA or HER2 antibodies) and have shown enhanced accumulation in prostate and breast tumors. SPIONs can likewise be conjugated with targeting molecules, and their magnetic cores allow external magnetic guidance to tumor sites. ZnO NPs have also been actively targeted, coating them with an antibody against a tumor angiogenesis marker (CD105) increased their tumor uptake. Nevertheless, even with active targeting, a considerable fraction of injected nanoparticles is sequestered by the liver and spleen, limiting the overall delivery to tumors. Radiolabeled Au, iron oxide, and ZnO nanoparticles have all demonstrated potent anti-cancer effects in animal models while simultaneously enabling imaging. AuNPs carrying therapeutic radionuclides (such as ^198^Au or ^177^Lu) have induced tumor regressions in mice and allowed parallel SPECT or PET imaging of the tumors. Similarly, SPION clusters dual-labeled with ^68^Ga (for PET) and ^177^Lu (for β^−^ therapy) provided concurrent tumour visualisation and radiotherapy, slowing tumour growth. ZnO NPs have shown excellent imaging capability for instance, ^64^Cu-ZnO enabled clear PET scans of tumors, and ZnO loaded with therapeutic nuclides could likewise deliver tumoricidal radiation before safely degrading.

Radiolabelled ZnO, iron oxide-based, and gold nanoparticles show strong potential for cancer imaging and therapy. Still, the field remains largely dominated by proof-of-concept studies rather than systematic design strategies. Future development should focus on how particle size, surface charge, coating chemistry, chelator stability, radionuclide half-life, tumour retention, and biological clearance collectively determine therapeutic performance and therapeutic index. Nanoparticles should be stable long enough to reach and irradiate tumour tissue, but should subsequently degrade, clear, or become biologically inactive to reduce long-term toxicity. Each material class offers distinct advantages and a few limitations: Au nanoparticles provide strong surface chemistry and radiolabelling versatility but may accumulate in the liver and spleen, ZnO nanoparticles are biodegradable but may dissolve prematurely, and iron oxide nanoparticles offer MRI visibility, magnetic responsiveness, and partial biodegradability, although reticuloendothelial accumulation remains a concern. Direct radionuclide incorporation into magnetic oxide lattices through isomorphic substitution or core-doping is a promising future direction, but current evidence is insufficient to treat it as an established replacement for chelator or coating-based radiolabelling. Overall, future systems should favour rational multifunctionality rather than excessive complexity and should include standardised evaluation of radiochemical stability, biodistribution, dosimetry, clearance, and therapeutic index.

In conclusion, radiolabeled inorganic nanoparticles armed with suitable chelators represent a cutting-edge approach in cancer theranostics, integrating nanotechnology potential with the proven success of radionuclide therapy. Our critical analysis of zinc oxide, iron oxide, and gold nanoparticle systems for prostate and breast cancer applications reveals that each platform brings unique advantages to the fight against cancer, as well as its own set of limitations. Gold nanoparticles distinguish themselves with facile surface chemistry, high radiolabeling versatility, and inert stability, enabling the creation of richly functionalized radiotheranostics (from ^198^Au nanoseeds for prostate cancer brachytherapy to antibody-targeted ^177^Lu-AuNPs for HER^2+^ breast cancer). Iron oxide nanoparticles offer an intrinsically biocompatible and multifunctional platform, one that not only ferries radioisotopes for imaging and therapy but also provides magnetic responsiveness for MRI diagnosis and hyperthermia treatment. Zinc oxide nanoparticles, while newer to the domain, emerge as a promising theranostic platform due to their biodegradability and potential for intrinsic cytotoxicity; they have successfully carried PET radiometals for tumor imaging and vascular targeting, and future studies are poised to exploit their combined diagnostic and therapeutic capabilities (e.g., in photodynamic therapy or drug delivery). At present, the field stands at an exciting yet preparatory stage: the current state of the art is that radiolabeled nanoparticles have shown compelling efficacy in animal models of breast and prostate cancer, achieving targeted tumor imaging and, in many cases, significant therapeutic tumor control. However, these advances have not yet translated into routine clinical practice. The challenges of ensuring in vivo stability, avoiding off-target toxicities, scaling up production, and navigating regulatory approval remain to be fully met. Proceeding onward, research efforts must concentrate on refining nanoparticle designs to improve tumor targeting specificity (potentially through multi-ligand approaches or stimulus-responsive systems) and on enhancing the clearance of these particles from non-target tissues to maximize safety. Promising future directions include the development of hybrid theranostic platforms for example, combining radiotherapy with photothermal or immune-stimulating elements in one NP construct and the use of alpha-emitting radionuclides for tackling micrometastatic disease with high potency when delivered by a nanoparticle vehicle. Additionally, extensive pharmacokinetic and long-term toxicity studies are needed for each candidate platform, to satisfy safety concerns and optimize dosing regimens. With continued interdisciplinary research, including materials science innovations and insights from nuclear medicine, radiolabeled inorganic nanoparticles are poised to overcome current limitations. In doing so, they could fulfil their potential as powerful theranostic agents, enabling clinicians to visualize and destroy tumors in tandem and offering patients with prostate or breast cancer a new generation of targeted radionanomedicine treatments. The evidence to date justifies cautious optimism: these nano-radiopharmaceuticals have shown that they can unite diagnosis and therapy in a single agent, and with further development, they hold the promise of improving treatment precision and outcomes in oncology.

## Figures and Tables

**Table 1:** Function-based and standardised comparison of radiolabelled ZnO, iron oxide-based, and Au nanoparticle systems for cancer imaging, biodistribution, and therapy.

Nanoparticle System	Synthesis/Surface Engineering	Physicochemical Properties	Drug/Ligand Loading or Functionalization	Radionuclide and Labelling Data	Stability Data	Biodistribution/Tumour Uptake	Therapeutic Efficacy/Biological Effect	Toxicity/Clearance Data	Critical Interpretation
NOTA-ZnO-PEG-TRC105	Red fluorescent ZnO NPs functionalized with PEG, NOTA, and TRC105 antibody.	Size/zeta: NR in extracted data.	TRC105 antibody for CD105 targeting; NOTA for ^64^Cu chelation.	^64^Cu/NOTA labelling; labelling and purification time 90 +/− 15 min; radiochemical yield 78.5 +/− 10.9%; purity > 95%; activity/ZnO ratio ~0.97 GBq/mg.	Radiochemical purity > 95% after purification; detailed serum stability NR.	4T1 tumour uptake: 6.4 +/− 0.8, 6.0 +/− 1.0, 5.1 +/− 0.6, 4.6 +/− 0.4 %ID/g at 0.5, 3, 16, 24 h. Non-targeted uptake: 1.8–2.5 %ID/g. Blocking reduced uptake to 1.7–2.8 %ID/g. Tumour-to-muscle ratio 12.1 +/− 2.6 at 3 h.	No tumour-growth inhibition or survival study.	No complete toxicity, histopathology, or long-term clearance. High liver/spleen uptake indicated RES clearance.	Strong ZnO imaging/targeting example; therapeutic relevance remains indirect.
^99m^Tc-CS-LC-ZnO nanoflakes	Chitosan-coated L-carnitine-loaded ZnO nanoflakes.	ZnO nanoflakes: crystallite size 48 nm; BET 32.85 m^2^/g; CS-LC-ZnONFS size 185–220 nm; PDI 0.151; zeta potential 25.9 +/− 6.4 mV.	L-carnitine loading 93.79 +/− 2.44%.	^99m^Tc direct labelling using SnCl_2_.2H_2_O; optimized yield 94.14 +/− 0.21% at pH 5, 150 ug/mL SnCl_2_, 30 min.	Stable in saline and rat serum up to 24 h.	Normal rats: blood retention 4.76 +/− 0.32 %ID/g at 24 h; testis uptake 9.97 +/− 0.41 %ID/g at 1 h, 7.46 +/− 0.14 %ID/g at 24 h.	Not a cancer model; no tumour uptake or anticancer efficacy.	No full toxicology or long-term clearance. Liver/kidney involvement observed.	Useful high-yield ZnO radiolabelling/biodistribution example, but not cancer-therapy evidence.
^99m^Tc-labelled DOX-loaded ZnO@dextran	Wet-chemical ZnO@dextran system loaded with DOX.	Hydrodynamic diameter 11.5 +/− 3 nm; PDI 0.18 +/− 0.01; zeta potential ~12 mV.	DOX loading efficiency 88%. pH-sensitive release: 7 +/− 1.17% at pH 7.4, 52.6 +/− 1.02% at pH 6, 93.1 +/− 1.6% at pH 5, 99.6 +/− 0.2% at pH 4.	^99m^Tc direct labelling using SnCl_2_; efficiency 98.5 +/− 1.3% at pH 7, 100 ug/mL SnCl2, 15 min.	Radiolabelling stability > 90% after 24 h in saline and plasma.	EAC tumour uptake 14 +/− 0.8 %ID/g at 1 h, decreasing to 2.8 +/− 0.6 %ID/g at 4 h. Tumour-to-non-tumour ratio 17.5 at 1 h.	EAC cells reduced to 3.2 × 10^7^, compared with 1.26 × 10^8^ for free DOX and 3.68 × 10^8^ for controls.	Hemolysis < 5% from 0.1–50 µg/mL. No complete histopathology, long-term toxicity, or clearance study.	Strongest ZnO theranostic example; combines radiolabelling, tumour uptake, pH-responsive DOX release, and tumour suppression.
[^177^Lu]Lu-MAPAD iron oxide NPs	Alginate/PEG-coated iron oxide NPs functionalized with DOX and bevacizumab.	MAPAD DLS size 474.6 +/− 13.8 nm.	Bevacizumab functionalization 41.84 wt.%; DOX loading 45.27 wt.%; DOX entrapment 82.67 +/− 3.87 wt.%.	^177^Lu direct labelling via alginate carboxylates; yield 93.41 +/− 2.59% after overnight incubation at 40 °C.	89.12 +/− 2.99% intact after 14 days at RT; serum stability decreased from 84.80 +/− 3.75% to 72.16 +/− 3.05% over 14 days.	Intratumoral tumour retention: 370.69 +/− 196.33% IA/g at 1 d, 92.50 +/− 29.45% IA/g at 2 d, 47.28 +/− 6.49% IA/g at 7 d. I.v. delivery mainly liver/spleen.	Single i.t. injection gave sustained tumour control over 50 days; TGI 0.76 +/− 0.72 at day 42, 1.10 +/− 0.63 at day 50.	Body-weight limits not exceeded. Slight bone uptake suggested possible radionuclide release.	Strong local nanobrachytherapy example; systemic delivery remains weak.
Radiolabelled iron oxide nanoflowers	Flower-like IONPs coated with citric acid, PAA, or PEG.	Individual core 13.5 +/− 1.2 nm; nanoflower diameter 24.8 +/− 4.4 nm; PAA@IONP SAR 478 W/g; ILP 7.3 +/− 0.2 nH m^2^/kg.	Coating comparison: CA, PAA, PEG.	PAA@IONP labelling yields: 97.5% ^99m^Tc, 99.5% 90Y, 99.7% ^177^Lu.	<10% detached ^99m^Tc after 24 h; <8% free 90Y after 72 h; >95% retained ^177^Lu after 96 h.	No animal biodistribution.	In vitro magnetic hyperthermia caused frequency/time-dependent CT-26 cell killing.	PAA@IONP non-toxic below 10 ug/mL; IC50 60 µg/mL. No in vivo toxicity.	Strong radiochemistry/hyperthermia feasibility study; lacks in vivo tumour validation.
^177^Lu-IONP-Folate	Clickable IONP-DBCO conjugated with folate and ^177^Lu-NOTA-N_3_.	IONP-DBCO hydrodynamic size 11.4 nm; zeta potential −9.6 mV.	Folate targeting; improved FR binding. K_d_ 10.68 +/− 2.53 nM vs. 52.74 +/− 8.26 nM for ^177^Lu-Folate.	^177^Lu/NOTA labelling; all three radiotracers showed 99% labelling efficiency.	NR.	48 h kidney uptake: 3.29 +/− 0.56%ID/g for 177Lu-IONP-Folate vs. 11.22 +/− 1.71%ID/g for 177Lu-Folate. Tumour absorbed dose 0.37 +/− 0.14 Gy/MBq.	No tumour-growth inhibition or survival.	Renal absorbed dose reduced from 2.46 +/− 0.50 to 1.01 +/− 0.17 Gy/MBq. Lung dose may be limiting.	Strong dosimetry study; supports kidney-dose reduction, not direct therapeutic efficacy.
^177^Lu-DMSA@SPIONs	Co-precipitated SPIONs coated with DMSA.	TEM size 11.4 +/− 3.2 nm; crystallite size 12.2 nm; DLS increased from 46.1 +/− 3.8 nm to 140.3 +/− 6.5 nm after DMSA; zeta shifted from +12.5 mV to −35.1 mV.	DMSA coating for colloidal stability and ^177^Lu binding.	^177^Lu direct labelling; yield 86.6 +/− 2.1%; purity after magnetic separation > 99%.	Up to 144 h; ^177^Lu^3+^ release 2.2 +/− 0.5% in suspension, 3.6 +/− 0.7% in serum, 4.2 +/− 1.0% in saline.	Intratumoral retention 90–95% ID at day 1; at day 14: ~92% ID in CT-26 and ~72% ID in 4T1.	Treated tumours ~2.5-fold smaller in CT-26 and ~5.5-fold smaller in 4T1 at day 14.	No significant body-weight differences; slight liver/kidney toxicity.	One of the strongest local iron oxide nanobrachytherapy studies.
^68^Ga-DOTA-IO-GUL	Amphiphile encapsulated iron oxide NPs bearing DOTA and PSMA-targeting GUL ligand.	Hydrodynamic diameter 11.01 +/− 1.54 nm.	PSMA-targeting GUL ligand.	^68^Ga/DOTA labelling at pH 5.6, 90 °C, 30 min; labelling efficiency and purity > 99%.	Serum stability 94% after 2 h; salt stability in 0.9–3.6% NaCl for 24 h.	PSMA-positive 22Rv1 tumour uptake; SUVmean 0.668 at 1 h. Blocking with MIP-1072 reduced uptake.	No therapy.	No full organ biodistribution, toxicity, histopathology, or long-term clearance.	Strong PSMA PET/MR imaging probe; therapeutic relevance hypothetical.
^99m^Tc-IONs-PSMA/BN	Co-precipitated IONs coated with MPTES silica shell and conjugated to PSMA and/or bombesin ligands.	DLS: IONs-SH 54.5 nm, IONs-PSMA 64.8 nm, IONs-BN 99.8 nm, IONs-PSMA/BN 66.5 nm. Zeta: −40.11, −30.38, −30.14, −10.15 mV, respectively.	PSMA ligand, bombesin ligand, or dual PSMA/BN.	Direct 99mTc labelling RCC: 95.05 +/− 2.26%, 95.95 +/− 1.63%, 96.18 +/− 1.98% for PSMA, BN, PSMA/BN.	Serum stability after 2 h: 98.99 +/− 0.30%, 96.36 +/− 0.55%, 98.05 +/− 0.33%. DTPA challenge after 24 h: 95.38–96.72% intact.	No animal biodistribution. In vitro receptor-associated binding in LNCaP/PC3 cells.	No therapy.	Hemolysis 0.0–0.1%; viability > 76% in PC3 and >71% in LNCaP at 20 µg/mL.	Good in vitro prostate cancer targeting proof-of-concept; requires in vivo validation.
^99m^Tc-DPA-alendronate-Endorem/Feridex SPIOs	Dextran-coated SPIOs labelled through bisphosphonate anchoring to Fe_3_O_4_ core.	Fe_3_O_4_ core 5 nm; hydrodynamic diameter 106 +/− 60 nm.	DPA-alendronate bisphosphonate anchor.	^99m^Tc-DPA-alendronate purity > 99%; optimized Endorem labelling ~55% after 15 min at 100 °C; 650 MBq/mg Fe.	Serum stability 96 +/− 2% at 24 h, 94 +/− 4% at 48 h; 100% retained in PBS at 48 h.	Healthy mice: liver 96.9 +/− 0.9% ID, spleen 1.3 +/− 0.4% ID at 1 h. Bone uptake 2 +/− 1%ID/g, supporting in vivo stability.	No tumour/therapy.	No formal toxicity or long-term clearance.	Important anchoring-method paper; not tumour therapy.
^64^Cu-DOTA-PEG-Au nanocages, 55 and 30 nm	Au nanocages PEGylated through Au-S anchoring and DOTA conjugation.	Edge lengths 54.5 +/− 4.4 nm and 30.3 +/− 4.2 nm; hydrodynamic diameters 96.0 +/− 12.0 nm and 63.7 +/− 7.3 nm; zeta 18.7 +/− 6.5 mV and 10.2 +/− 1.1 mV.	PEG coverage 45,000 chains/AuNC for 55 nm and 17,000 for 30 nm.	^64^Cu/DOTA labelling; specific activity 81.4–107.3 GBq/nmol.	Serum stability 90.2 +/− 0.3% at 4 h, 81.5 +/− 1.4% at 24 h.	30 nm AuNC tumour uptake: 2.68 +/− 0.12%ID/g at 1 h, 7.2 +/− 0.9 at 4 h, 7.9 +/− 1.1 at 24 h; tumour-to-muscle 25.7 +/− 6.9 at 24 h.	No therapy.	No full toxicity or long-term clearance; liver uptake remained substantial.	Strong size-dependent PET pharmacokinetic study; therapeutic benefit not proven.
^64^Cu-NOTA-Au-IONP-Affibody	Dumbbell Au-Fe_3_O_4_ heterostructure; Au side for NOTA/^64^Cu, Fe_3_O_4_ side for MRI and Affibody conjugation.	Au domain ~4 nm; Fe_3_O_4_ domain ~8 nm; hydrodynamic diameter increased from 19.8 +/− 1.8 nm to 24.4 +/− 2.0 nm; zeta ~−25 to −30 mV.	213 +/− 47 Affibody molecules and 13.7 +/− 2.4 NOTA chelators per particle.	^64^Cu/NOTA labelling yield ~60%; specific activity 74–222 MBq/nmol particle.	Colloidally stable in 10% FBS/PBS at 37 °C for 48 h.	A431 tumour uptake ~3.5%ID/g at 4 h, 4.6%ID/g at 24 h; blocking reduced to 1.9%ID/g. MRI tumour signal drop 44.0% at 48 h.	No therapy.	No formal toxicity/clearance; high liver/spleen uptake.	Strong EGFR-targeted multimodal imaging study; diagnostic rather than therapeutic.
^99m^Tc-Dox-EGCG-AuNPs	EGCG-stabilized AuNPs loaded with radiolabelled DOX.	DLS increased from 21.4 +/− 1 nm to 58.9 +/− 2 nm after DOX loading.	DOX loading: 0.4 mg DOX used.	^99m^Tc-DOX yield 93.5 +/− 2.04% using 1 mg DOX, 100 ug SnCl_2_, pH 7, 15 min.	^99m^Tc-Dox stable up to 6 h; 99mTc-Dox-EGCG-AuNPs reportedly stable in saline/rat serum for 3 days.	Ehrlich tumour uptake after i.v. injection 22.45%ID/g at 2 h. I.t. retention 80.22%ID/g at 15 min, decreasing to ~44–45%ID/g at 120 min.	In vitro cytotoxicity improved: MCF-7 IC50 40.78 +/− 1.40 µL/well; HepG-2 IC50 24.4 +/− 2.35 uL/well.	No tumour-growth inhibition, survival, systemic toxicity, cardiac toxicity, or long-term clearance. Non-peer-reviewed preprint version.	Preliminary radiotracking/cytotoxicity study; therapeutic efficacy not demonstrated.
^99m^Tc/^177^Lu-AuNP-Tat-BN	5 nm AuNPs functionalized with Tat-BN, ^177^Lu-DOTA-GGC, and ^99m^Tc-HYNIC-TOC.	Final hydrodynamic diameter 8.07 +/− 3.77 nm; zeta −48 +/− 4.6 mV.	Per AuNP: ~8 Tat-BN, ~10, ^177^Lu-DOTA-GGC, ~10 ^99m^Tc-HYNIC-TOC.	Final radiochemical purity 96 +/− 2% without purification; specific activity 50 MBq/nmol.	NR.	No animal data. PC3 internalization 19.85 +/− 1.10%, reduced to 12.11 +/− 1.46% after blocking.	Laser 532 nm, 0.65 W/cm^2^, 6 min reduced PC3 viability to 1.3 +/− 0.4% with AuNP-Tat-BN. Dual-labelled system inhibited PC3 proliferation over 3 days.	No in vivo toxicity, biodistribution, dosimetry, or clearance.	Strong in vitro multifunctional therapy concept; in vivo validation missing.
^177^Lu-AuNP-c(RGDfK)C	20 nm AuNPs functionalized through Au-S interactions with c(RGDfK)C and ^177^Lu-DOTA-GGC.	Citrate AuNP diameter 18.64 +/− 0.13 nm; functionalized AuNP diameter 20.33 +/− 0.07 nm.	~108 c(RGDfK)C and ~270 ^177^Lu-DOTA-GGC molecules per AuNP.	Final purity 96 +/− 2% without purification; 92 +/− 2% after 8 days.	Radiochemical purity remained 92 +/− 2% after 8 days.	U87MG tumour uptake 2.51 +/− 0.52%ID/g at 1 h, 6.42 +/− 0.71%ID/g at 6 h, 2.14 +/− 0.37%ID/g at 96 h. Blocking reduced 1 h uptake to 1.32 +/− 0.24%ID/g.	No tumour-growth inhibition. Tumour absorbed dose 0.357 +/− 0.052 Gy/MBq, higher than dimer 0.252 +/− 0.027 and monomer 0.102 +/− 0.018 Gy/MBq.	No toxicity, body-weight, histopathology, or clearance study.	Strong biodistribution/dosimetry support for multivalent RGD-AuNPs; therapeutic benefit remains dosimetric, not proven.
[^225^Ac]Ac-Au@TADOTAGA	Ultrasmall AuNPs coated with TADOTAGA chelator shell.	Au core 2–3 nm; hydrodynamic diameter 5–9 nm; colloidally stable across pH 3–11; negative zeta potential above pH 4.	TADOTAGA provides thioctic-acid Au anchoring and DOTA-like ^225^Ac chelation.	225Ac labelling at pH 5.6, 70 °C, 30 min; yield 86 +/− 1.8%; recovery 68.5 +/− 2.3%; purity > 93%.	After 10 days: ~78% stable in PBS and ~84% in acetate buffer.	U87MG i.v. tumour uptake 4.05 +/− 0.34%IA/g at 2 h. I.t. retention 60.67 +/− 3.87%IA/g at 2 h, decreasing to 5.21 +/− 1.26%IA/g at 288 h.	Three i.t. injections totaling ~15 kBq/mouse delayed tumour growth; TGI ~2.4-fold lower at day 8, ~3.9-fold lower at day 22. Necrosis significantly increased, *p* < 0.0001.	Non-labelled AuNPs relatively non-toxic. Radiolabelled system reduced U87MG viability to 22% at 24 h and 13% at 48 h at 2 kBq/mL. Bone uptake increased after i.v. injection.	Strong Au alpha-nanobrachytherapy proof-of-concept; systemic use limited by kidney/liver/spleen/bone uptake and daughter retention concerns.
^177^Lu-MCP-AuNPs: OPSS, LA, PEG-pGlu(DOTA)8-LA4	30 nm AuNPs modified with radiolabelled PEG metal-chelating polymers via monothiol, dithiol, or multithiol Au-S anchoring.	Unmodified AuNP Dh 37.8 +/− 4.2 nm; OPSS-AuNP 56.6 +/− 14.2 nm; LA-AuNP 54.2 +/− 14.0 nm; multithiol AuNP 46.0 +/− 8.4 nm.	Polymer molecules per AuNP: 965 +/− 96 OPSS, 578 +/− 63 LA, 847 +/− 74 multithiol.	^177^Lu labelling efficiency > 85%; final purity > 99%.	Human plasma, 72 h: multithiol retained 59.2 +/− 8.4% bound to AuNPs vs. 17.6 +/− 3.1% OPSS and 16.6 +/− 4.3% LA.	No tumour model. At 168 h, eliminated radioactivity: 61.4 +/− 3.5% ID OPSS, 44.8 +/− 6.4% ID LA, 38.3 +/− 2.4% ID multithiol. Liver uptake lowest for multithiol 25.0 +/− 2.0% ID, spleen highest 18.7 +/− 6.3% ID.	No therapy.	Urinary excretion accounted for >90% of eliminated radioactivity. Au and ^177^Lu distributions were not always identical.	Mechanistic stability/biodistribution study; shows anchoring chemistry controls radiolabel fate but does not prove therapeutic efficacy.

## Data Availability

No new data were created or analyzed in this study. Data sharing is not applicable to this article.

## Abbreviations

*Au*	Gold
*AuNCs*	Gold Nanocages
*BFC*	Bifunctional Chelator
*BN*	Bombesin
*DCM*	Dichloromethane
*DEX*	Dextran
*DFO*	Desferrioxamine
*DMF*	Dimethylformamide
*DOTA*	1,4,7,10-Tetraazacyclododecane-1,4,7,10-Tetraacetic Acid
*DOTAGA*	DOTA-Aza-Macrocycle Derivative
*DOX*	Doxorubicin
*DPA*	Dipicolylamine
*DSB*	Double-Strand Break
*DTPA*	Diethylenetriamine Pentaacetic Acid
*EBRT*	External Beam Radiotherapy
*EDC*	1-Ethyl-3-(3 dimethylaminopropyl)carbodiimide
*EGCG*	Epigallocatechin Gallate
*EPR*	Enhanced Permeability and Retention
*FDA*	Food and Drug Administration
*GRP*	Gastrin-Releasing Peptide
*HYNIC*	Hydrazinonicotinamide
*IONPs*	Iron Oxide Nanoparticles
*LA*	Lipoic Acid
*MCP*	Metal Chelating Polymer
*NHS*	N-Hydroxysuccinimide
*NOTA*	1,4,7-Triazacyclononane-1,4,7-Triacetic Acid
*NPs*	Nanoparticles
*OPSS*	Ortho-Pyridyl Disulfide
*PBS*	Phosphate-Buffered Saline
*PEG*	Polyethylene Glycol
*PGlu*	Polyglutamic Acid
*PSMA*	Prostate-Specific Membrane Antigen
*ROS*	Reactive Oxygen Species
*RT*	Room Temperature
*SnCl_2_*	Stannous Chloride
*SPIONs*	Superparamagnetic Iron Oxide Nanoparticles
*SSB*	Single-Strand Break
*TADOTAGA*	Tetraazacyclododecane Tetraamide Derivative
*TCEP*	Tris(2-Carboxyethyl)Phosphine
*TCPP*	meso-Tetrakis(4-Carboxyphenyl)Porphyrin
*TLC*	Thin-Layer Chromatography
*ZnO*	Zinc Oxide
